# Kidney-derived c-kit^+^ progenitor/stem cells contribute to podocyte recovery in a model of acute proteinuria

**DOI:** 10.1038/s41598-018-33082-x

**Published:** 2018-10-03

**Authors:** Erika B. Rangel, Samirah A. Gomes, Rosemeire Kanashiro-Takeuchi, Russell G. Saltzman, Changli Wei, Phillip Ruiz, Jochen Reiser, Joshua M. Hare

**Affiliations:** 1 Interdisciplinary Stem Cell Institute, Leonard M Miller School of Medicine University of Miami, Miami, 33136 Florida USA; 2 Hospital Israelita Albert Einstein Hospital, São Paulo, 05652 São Paulo, Brazil; 3 Federal University of São Paulo, São Paulo, 04023 São Paulo, Brazil; 4 Laboratory of Cellular, Genetic, and Molecular Nephrology, Renal Division, University of São Paulo, 01246 São Paulo, Brazil; 5 Department of Molecular and Cellular Pharmacology, Leonard M Miller School of Medicine, University of Miami, Miami, 33136 Florida USA; 6 Department of Medicine, Rush University Medical Center, Chicago, 60612 Illinois USA; 7 Departments of Surgery and Pathology, Leonard M Miller School of Medicine University of Miami, Miami, 33136 Florida USA; 8 Division of Cardiology, Leonard M Miller School of Medicine University of Miami, Miami, 33136 Florida USA

## Abstract

Kidney-derived c-kit^+^ cells exhibit progenitor/stem cell properties and can regenerate epithelial tubular cells following ischemia-reperfusion injury in rats. We therefore investigated whether c-kit^+^ progenitor/stem cells contribute to podocyte repair in a rat model of acute proteinuria induced by puromycin aminonucleoside (PAN), the experimental prototype of human minimal change disease and early stages of focal and segmental glomerulosclerosis. We found that c-kit^+^ progenitor/stem cells accelerated kidney recovery by improving foot process effacement (foot process width was lower in c-kit group *vs* saline treated animals, *P* = 0.03). In particular, these cells engrafted in small quantity into tubules, vessels, and glomeruli, where they occasionally differentiated into podocyte-like cells. This effect was related to an up regulation of α-Actinin-4 and mTORC2-Rictor pathway. Activation of autophagy by c-kit^+^ progenitor/stem cells also contributed to kidney regeneration and intracellular homeostasis (autophagosomes and autophagolysosomes number and LC3A/B-I and LC3A/B-II expression were higher in the c-kit group *vs* saline treated animals, *P* = 0.0031 and *P* = 0.0009, respectively). Taken together, our findings suggest that kidney-derived c-kit^+^ progenitor/stem cells exert reparative effects on glomerular disease processes through paracrine effects, to a lesser extent differentiation into podocyte-like cells and contribution to maintenance of podocyte cytoskeleton after injury. These findings have clinical implications for cell therapy of glomerular pathobiology.

## Introduction

Injury and loss of podocytes are leading factors of glomerular disease and renal failure. Podocytes are specialized epithelial cells located on the visceral side of the glomerulus that play a critical role in the renal glomerular filtration barrier via their foot processes^1^. Notably, podocytes are terminally differentiated cells that do not undergo cell division. The search for putative stem cells or precursors within the kidney is the focus of extensive research, and their role in renal homeostasis and regeneration has been recently highlighted^2,3^.

Bone marrow-derived progenitor cells do not contribute to podocyte turnover, as reported in different models of glomerular injury in rats^4^. In the human adult kidney, a hierarchical population of progenitors organized in a precise sequence along Bowman’s capsule has also been identified^5^. That population of CD24^+^CD133^+^ renal progenitors was found to be a reservoir of cells that may contribute to the turnover of senescent or injured podocytes by proliferating, migrating and differentiating from the urinary to the vascular stalk^5^. However, genetic labelling of parietal epithelial cells (PECs) documented that their recruitment occurs mainly in juvenile mice^6^ and only a small fraction of these cells are recruited to glomeruli in adult mice^7^. Likewise, PECs were not involved in podocyte regeneration in models of glomerular hypertrophy in adult animals^7^, although these cells were identified as a possible source of regenerating podocytes in diabetic nephropathy^8^ or after treatment with glycogen synthase kinase 3-α and 3-ß (GSK3s) inhibitor^9^. Of importance, abnormal proliferation of these renal progenitors of the Bowman’s capsule can generate hyperplastic glomerular lesions and scarring in collapsing glomerulopathy and crescentic glomerulonephritis^10^.

Stem cells obtained from extra-renal sites, such as whole bone marrow (BM), BM-derived mesenchymal stem cells (BM-MSCs) and amniotic fluid stem cells (AFSCs), have been tested for podocyte regeneration. Although BM-MSCs can improve interstitial fibrosis and inflammation, and the loss of peritubular capillaries, they failed to modify proteinuria and prevent the progression of chronic kidney disease in different animal models of glomerular injury^11–15^. Moreover, beneficial effect of MSCs can be offset by a long-term adipogenic maldifferentiation accompanied by glomerulosclerosis^16^, whereas autologous hematopoietic stem cells can contribute to angiomyeloproliferative lesions in native kidneys^17^. Although engraftment was not the main mechanism of kidney regeneration, AFSCs^18^ and whole BM^19,20^ delayed interstitial fibrosis and progression of glomerulosclerosis, and increased animal survival.

We recently documented that kidney-derived c-kit^+^ cells represent a novel population of progenitor/stem cells with tubular regenerative capacity^21^. In a model of acute ischemia-reperfusion injury, c-kit^+^ progenitor/stem cells engrafted into tubules and glomeruli. We hypothesized therefore whether kidney-derived c-kit^+^ progenitor/stem cells have the potential to restore podocyte function in a model of acute proteinuria induced by puromycin aminonucleoside (PAN) in rats. Due to anti-inflammatory, anti-fibrotic and immune-modulatory properties rather than cellular differentiation of BM-MSCs, these cells were also injected in PAN model as a positive control of stem cell treatment.

PAN model is an experimental prototype of human minimal change disease (MCD) and early stages of focal and segmental glomerulosclerosis (FSGS)^22^. MCD is the most common cause of nephrotic syndrome in children (70–90%) and accounts for 10 to 15% of nephrotic syndrome in adults. PAN model is characterized by massive proteinuria (peak at day 8 and normalization at 4 weeks), reversible foot process effacement (FPE), podocyte detachment from the glomerular basement membrane, cytoskeletal changes, and podocyte apoptosis^22^.

Autophagy is a key homeostatic mechanism to maintain podocyte integrity. Genetic deletion of autophagy-related genes lead to proteinuria^23^. To note, PAN injection is associated with autophagy disruption and when autophagy is restored, podocyte damage started recovering^24^. Moreover, tight balance of mTOR (mammalian target of rapamycin) activity is crucial for podocyte homeostasis. mTORC1-Raptor regulates autophagy, whereas mTORC2-Rictor is important for cell survival, metabolism, proliferation, and cytoskeleton organization^25^. Genetic deletion of mTORC1 in podocyte lead to proteinuria and glomerulosclerosis. That progression is even worsened by simultaneous deletion of mTORC2^26^. Conversely, in diabetic nephropathy, genetic deletion or reduced activity of mTORC1 prevented glomerulosclerosis^26,27^.

Here, we tested the hypothesis that kidney-derived c-kit^+^ progenitor/stem cells exhibit multi-compartment engraftment, including the glomeruli, vascular and tubule-interstitial renal compartments. We further identify that these cells preserve podocyte cytoskeleton by modulating mTOR-Rictor pathway and autophagy after PAN injection. Therefore, our findings have implications for developing novel therapeutic applications for glomerular pathologies.

## Material and Methods

### Stem cell isolation and expansion

#### All experiments were performed in accordance with relevant guidelines and regulations

Bone marrow-derived mesenchymal stem cells were isolated from male 2 month-old GFP-Sprague-Dawley (SD) rats (Charles River, Wilmimgton-Massachusetts, USA) and kidney-derived c-kit^+^ cells were isolated from neonatal Sprague -Dawley rats and labelled with GFP-lentivirus, as previously described^21^ and also in the Supplementary Materials. Briefly, neonatal rat kidneys from Sprague-Dawley (SD; n = 6–8) were harvested, chopped, digested with collagenase II, and incubated in red blood cell lysing buffer (Sigma-Aldrich, St. Louis, MO, USA). Expansion medium included Dulbecco’s Modified Eagle Medium (DMEM/F12), 20% fetal bovine serum (FBS), 100 U/ml penicillin and 100 µg/mL streptomycin (Sigma-Aldrich, St. Louis, MO, USA). C-kit^+^ cells were isolated by immunopanning using rabbit polyclonal c-kit antibody (H300, Santa Cruz, Dallas, TX, USA) and further selected using FACS (BD FACSAria^TM^, University of Miami), as described in detail previously^21^. Depletion of hematopoietic stem cells lineage was also performed to ensure that c-kit^+^ cells came from kidney and that bone marrow-derived cells were removed. Therefore, the APC lineage antibody cocktail was used, which depletes CD3e, CD11b, CD45R/B220, erythroid cells, and Ly-6G and Ly-6C (BD Pharmigem, San Jose, CA, USA), and the anti-mouse CD117-PE conjugated (eBioscience, San Diego, CA, USA). Sorted C-kit^+^/Lin^−^ cells were plated and cultured in DMEM/F12 supplemented with 10% FBS, 10 ng/mL bFGF, 20 ng/mL EGF, 40 ng/mL SCF (stem cell factor) (PeproTech, Rocky Hill, NJ, USA), 10 ng/mL LIF (leukemia inhibitory factor; Millipore, Billerica, MA, USA), ITS (insulin-transferrin-selenium A liquid media supplement; Invitrogen, Carlsbad, CA, USA), and antibiotics, as documented in detail previously^21^. Cells were splited when reached ~80% of confluence. Medium was changed every other day. All cells were cultured at 37 °C in 98% humidified air containing 5% CO_2_.

### Puromycin aminonucleoside (PAN) administration and suprarenal stem cell or saline injection

The respective Institutional Animal Care and Use Committees of the University of Miami approved all procedures involving animals (number 10–176, Leonard M Miller School of Medicine University of Miami, Miami, Florida). For all experiments, female two month-old SD rats weighing 200–350 g (Charles River, Wilmimgton-Massachusetts, USA) were injected with a single dose of PAN (15 mg/100 g body weight; Sigma-Aldrich, St. Louis, MO, USA) via intra-peritoneal route. GFP-labelled c-kit^+^ cells or MSCs from GFP-SD (2 × 10^6^ cells) or saline were injected directly into the suprarenal aorta, as previously described^21^. Briefly, GFP- labelled c-kit^+^ cells or MSCs from GFP-SD or saline were injected directly into the abdominal aorta above the renal arteries, after application of a vascular clamp to the abdominal aorta below the renal arteries, as previously described^21^. Cells were resuspended in 300 µl of saline. The same volume of saline (300 µl) was injected into aorta in the control group. The needle used for cell injection was 31G, length 8 mm (5/16″) insulin syringe needle (BD Biosciences, San Jose, CA, USA. Rats were checked twice per day in the first 48 h after the procedure and then once per day. Analgesia was performed with buprenorphine via subcutaneous route. No animal died or became severely ill during the protocol, yet we were in accordance with the rules of incorporating humane endpoints for treatment or for early intervention in case of pain or distress. At the end of the protocol, animals were euthanized under anesthesia (Isofluorane) followed by exsanguination.

Kidneys were harvested 10 or 21 days after PAN injection for histological analyses. In the first analysis, animals treated with saline (n = 5) or kidney-derived c-kit^+^ progenitor/stem cells (n = 8) were sacrificed at day 10 after PAN injection. At day 10, the hypothesis was to verify whether kidney-derived c-kit^+^ progenitor/stem cell treatment was effective in reducing the peak of proteinuria after PAN injection. Since these cells did not contribute to a decrease in proteinuria levels, we moved to the second analysis to verify if progenitor/stem cells were able to accelerate kidney recovery. In that analysis, animals were treated with saline (n = 12), kidney-derived c-kit^+^ progenitor/stem cells (n = 10) or MSCs (n = 6). Therefore, we have data for MSC treatment exclusively in the second analysis, e.g., at day 21, and not at day 10. Furthermore, due to paracrine properties rather than cellular differentiation of MSCs, these cells were also injected in PAN model as a positive control of stem cell treatment. However, after injecting those six animals, we did not observe a significant change in functional kidney parameters at day 10 and then we decided therefore to include only the time-point of 21 days for molecular analyses in MSC-treated group. At day 21, kidney-derived c-kit^+^ progenitor/stem cell group was compared to saline and MSC-treated groups in order to analyze whether progenitor/stem cell treatment conferred advantage of accelerating kidney recovery after PAN injection.

### Blood collection and urinalysis

Blood collection was performed at baseline and days 5, 10, 14, and 21-post PAN injection for creatinine and blood urea nitrogen (BUN) measurement (Products Vitros Chemistry, Rochester, NY, USA). Albuminuria was analyzed by a dipstick (DiaScreen, Fisher Scientific, Pittsburgh, PA, USA) for proteinuria confirmation and then measured by a standard colorimetric assay (QuantiChrom^TM^ BCG Albumin Assay Kit; BioAssay Systems, Hayward, CA, USA) at those time-points. Animals that did not develop proteinuria assessed by a dipstick at day 4 were not included in the study. Absorbance was measured at 620 nm. Albumin levels were normalized to urine creatinine. Results were expressed as urine albumin-to-creatinine ratio (UACR; g/mg).

### Histological analyses and morphological studies on kidney following PAN injection

Kidneys were harvested 10 or 21 days after PAN injection for histological analyses. Fixed kidney specimens embedded in paraffin were sectioned at 4–5 μm thickness and stained with hematoxylin and eosin and periodic acid-Schiff reagent (PAS; Sigma-Aldrich, St. Louis, MO, USA) to evaluate glomerular and tubule-interstitial damage, which was characterized by acute tubular necrosis, cast formation, and interstitial inflammation, and mesangial expansion. Acute tubular necrosis (ATN) was assigned by semi-quantitative analysis of each individual variable (brush border loss, tubular dilation, necrosis, and calcification), as follows: 0 = normal histology; 1 = up to one-third of tubular cross section showing one of those variables; 2 = greater than one-third and less than to two-thirds of tubular cross section showing one of those variables; 3 = greater than two-thirds of tubular cross section showing one of those variables. The average was pooled to the presence of tubular casts (0–3), mesangial expansion (0–3) and interstitial infiltration (0–3) to augment the injury score (maximum 12), as reported in detail previously^21^. A blinded pathologist (P.R.) performed the analyses.

### Immunofluorescence confocal microscopy

For immunofluorescence, renal sections were embedded in Optimal Cutting Temperature compound (OCT, Sakura; Torrance, CA, USA) and subsequently frozen. Cryosections were then blocked for 1 h in a solution of 10% donkey serum and 0.1–0.3% Triton-X (Sigma-Aldrich, St. Louis, MO, USA). Primary antibodies were applied overnight at 4 °C, and included rabbit polyclonal anti-α-Actinin-4 and anti-laminin, mouse monoclonal or rabbit polyclonal anti-aquaporin 1 (Abcam, Cambridge, MA, USA), rabbit polyclonal anti-WT-1 and anti-VEGF (Santa Cruz, Dallas, TX, USA), mouse monoclonal anti-synaptopodin (Progen, Heidelberg, Germany), mouse monoclonal anti-smooth muscle actin (Sigma-Aldrich, St. Louis, MO, USA). Incubations for 1 hour using 488 or 568-conjugated secondary antibodies (Invitrogen, Carlsbad, CA, USA) were then performed. For GFP staining, rabbit or goat polyclonal anti-GFP FITC-conjugated (Abcam, Cambridge, MA, USA) were applied overnight at 4 °C. Nuclei labeling was obtained with DAPI (4′,6-diamidino-2-phenylindole). In control experiments, the primary antibody was omitted. Scoring for WT-1-positive cells was carried out by counting the number of positive nuclei in four randomly chosen kidney cortex and outer medulla sections using X10 magnification and after applying a rabbit polyclonal WT-1 antibody (Santa Cruz, Dallas, TX, USA). Data from all fields and all kidneys were pooled to obtain WT-1 score. Alpha actinin-4 was scored from 0 to 4+: 0, no staining; 1+, staining of <25% of the glomerular tuft area; 2+, 3+ and 4+ for 25–50, 51–75 and >75%, respectively, of podocytes of the glomerular tuft area staining^28^.

### Transmission electron microscopy (TEM) and measurements of foot process effacement (FPE)

Detailed protocols of immunofluorescence and TEM are described in the Supplementary Materials and as previously described^29^. Ultrathin sections were made of one or two glomeruli per tissue specimen. Ten to 15 photographs, covering one or two glomerular cross-sections, were made with a Philips CM10 transmission electron microscope (FEI, Hillsboro, OR, USA) at Miller School of Medicine, University of Miami. Briefly, quantified foot process width of each sample was taken from 3–4 glomeruli.

With the use of Image J 1.26t software (National Institutes of Health, rsb.info.nih.gov/ij), GBM was traced and measured from each picture. The number of podocyte foot processes along the GBM was counted by hand. A foot process was defined as any connected epithelial segment butting on the basement membrane, between two neighboring filtration pores or slits. Then, the arithmetic mean of the foot process width (FPW) was calculated by dividing the total GBM length per total foot process counted in each picture. The correction factor π/4 serves to correct for the random orientation in which the foot process are sectioned. A mean GBM length of 130 μm was evaluated in each glomerulus. For each animal, the mean FPW was calculated and that value was used to finally calculate a mean FPW for each group.

### RNA extraction and quantitative real-time PCR

Total RNA was isolated from renal cortices and cDNA was synthesized as described. TaqMan Universal PCR Master Mix (Applied Biosystems, Waltham, MA, USA) and primers/probes sets for specific genes (Applied Biosystems, Waltham, MA, USA) were used for real-time PCR (iQ5 multicolor real-time PCR detection system; Bio-Rad, Hercules, CA, USA). Absolute number of transcripts was calculated by 2^ΔCt^ method.

### Protein extraction and western blot analyses

Renal cortices were homogenized in an ice-cold buffer containing a protease inhibitor cocktail. To note, whole kidney cortex was collected for both RNA and protein analyses instead of glomerular lysates because bead perfusion would affect histological analyses of GFP-labelled cells. Protein lysates were prepared in RIPA buffer and quantified using Bradford Assay (Bio-Rad,﻿ #5000001). Samples were resolved by 3–8%, 7% or 10% SDS-PAGE and transferred to nitrocellulose or PVDF membranes (Bio-Rad, Hercules, CA, USA). The membranes were incubated with the antibodies indicated below, washed, and incubated with horseradish peroxidase-coupled secondary antibodies (Cell Signaling, Danvers, MA, USA). Prior to blocking and antibody incubation, membranes were stained with Ponceau S reagent (Sigma-Aldrich, #P3504) to visualize protein bands and cut at specific sizes so different antibodies could be tested at the same time. Blots were visualized using an enhanced chemiluminescence detection system (Bio-Rad, Hercules, CA, USA). Primary antibodies used in this study included rabbit polyclonal against LC3 A/B-I and II (#4108, Cell Signaling, Danvers, MA, USA), rabbit monoclonal against Raptor, Rictor, mTOR, GβL, phospho mTOR Ser 2448, and phospho mTOR Ser 2481 (all from Cell Signaling, mTOR pathway antibody sampler kit # 9964, Danvers, MA, USA), rabbit polyclonal anti-WT-1 (# sc-192) and anti-GAPDH (# sc-32233) (all from Santa Cruz, Dallas, TX, USA). Densitometry analysis of western blots was performed using Quantity One software (Bio-Rad Laboratories). The final blots correctly represent the original data and conform to community standards.

### Statistical analyses

Results are expressed as the mean ± SEM. The means of two populations were compared by Student’s *t*-test or Mann-Whitney test. For multiple comparisons, analysis of variance was employed followed by Tukey’s multiple comparison test (95% of confidence interval). A *P* value < 0.05 was considered statistically significant.

## Results

### Kidney-derived c-kit^+^ progenitor/stem cells accelerated urine albumin-to-creatinine ratio (UACR) improvement and ameliorated foot process effacement after PAN injection

**Figure 1:**
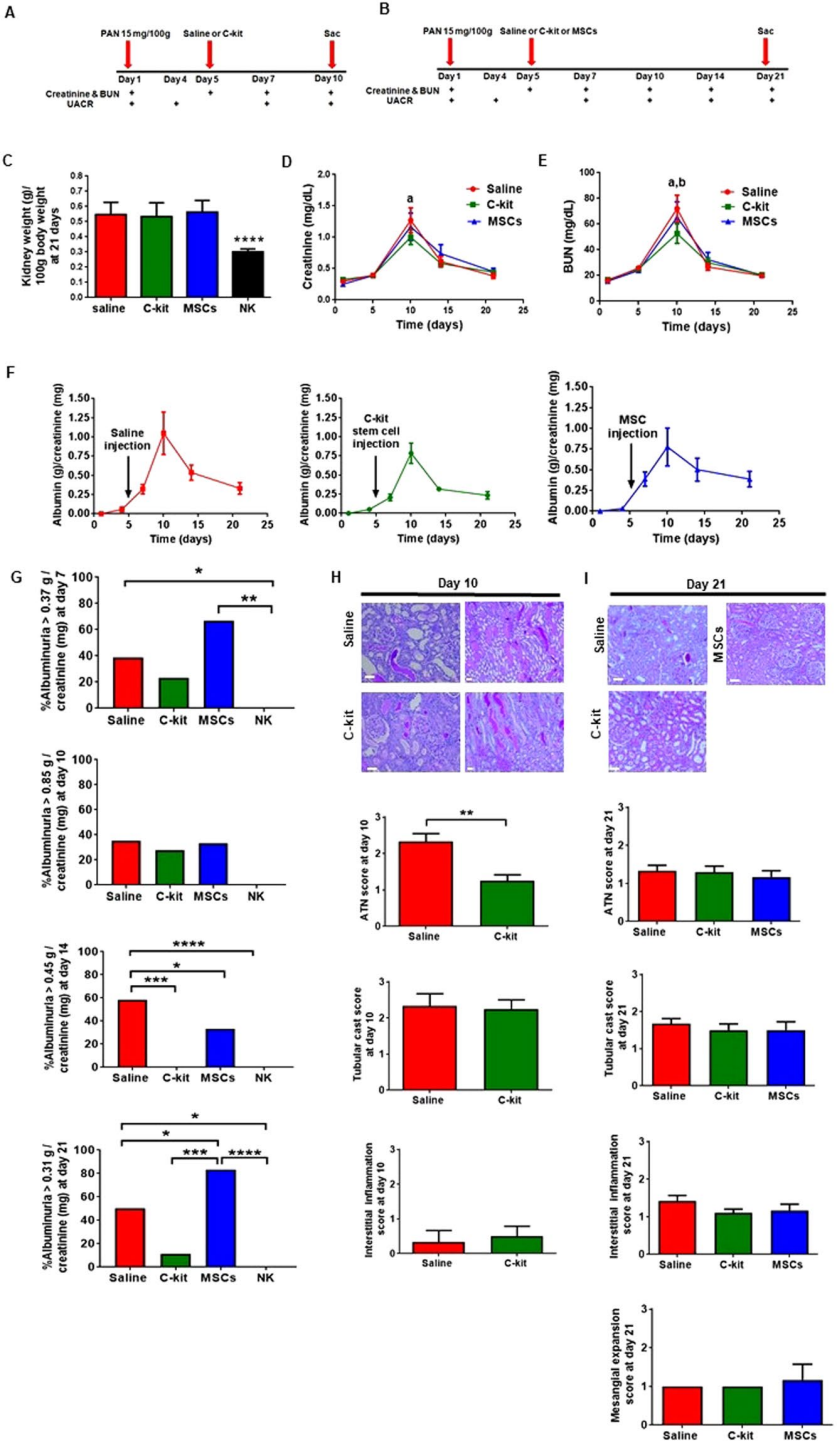
Functional and morphological changes in the saline, c-kit, and MSC treated groups according to the time. (**A**,**B**) Schemes of the procedure. Serum creatinine and blood urea nitrogen (BUN) and urine albumin-to-creatinine ratio (UACR) were measured at different time-points, e.g., 10 days (**A**) or 21 days (**B**). (**C**) Stem cell therapy did not avoid kidney weight increase after PAN injection. Kidney weight (g)/100 g body weight was higher in all three groups when compared to the normal kidney (****P < 0.0001). (**D**) Serum creatinine levels were lower in the c-kit treated group in comparison to the saline group at day 10 (a, *P* = 0.0043), although no difference was observed in all three groups at day 21. (**E**) BUN levels were lower in the c-kit treated group when compared to the saline (a, *P* = 0.0002) and MSC (b, *P* = 0.037) treated groups at day 10, although no difference was observed in all three groups at day 21. (**F**) Time course assessment of UACR in all three groups according to the time. No significant differences were observed by analysis of variances. (**G**) At days 7, 10, 14 and 21, overall average of UACR was 0.37, 0.85, 0.45 and 0.31 g/creatinine (mg), respectively. At day 7, saline (~40%) and MSC (~67%)-treated groups exhibited higher UACR values when compared to normal kidneys (NK) (*P = 0.039 and **P = 0.0031, respectively). At day 10, there was no difference between treated groups and NK. Saline-treated group (~60%) exhibited higher UACR values when compared to NK (****P < 0.0001), MSC-treated group (~17%; *P = 0.038) and c-kit treated group (0%; ***P = 0.0002) at day 14. Saline treated group (~33%) maintained higher UACR values when compared to NK (*P = 0.04), but lower than MSC-treated group (~80%; *P = 0.018) at day 21. C-kit treated group (10%) exhibited higher UACR values when compared to NK (****P < 0.0001), but lower UACR values when compared to MSC-treated group (***P = 0.0004) at day 21. (**H**) Representative of periodic acid-Schiff (PAS) staining of renal cortex sections in the saline, c-kit, and MSC treated groups. Semi-quantitative injury score was assessed by the presence of acute tubular necrosis (ATN), tubular cast formation, and interstitial inflammation. ATN score was lower in the c-kit treated group when compared to the saline group (**P = 0.0014) at day 10. No mesangial expansion was observed at this time-point. (**I**) Representative of periodic acid-Schiff (PAS) staining of renal cortex sections in all three groups at day 21. Semi-quantitative injury score was assessed by the presence of acute tubular necrosis (ATN), tubular cast formation, interstitial inflammation, and mesangial expansion. No difference was observed. All data are means ± SEM. Scale bars represent 50 μm for (**H**) and (**I**). At day 10, n = 5 and n = 8 for saline and c-kit-treated group, respectively, and n = 12, n = 10, and n = 6 for saline, c-kit, and MSC-treated groups at day 21, respectively.

Figure 1A,B describe the schemes of PAN administration. In all experiments, PAN was injected at day 1, proteinuria was measured at day 4, and the rats were randomized according to treatment at day 5. In the first analysis, animals treated with saline (n = 5) or kidney-derived c-kit^+^ progenitor/stem cells (n = 8) were sacrificed at day 10 after PAN injection (Fig. 1A). In the second analysis the animals were treated with saline (n = 12), kidney-derived c-kit^+^ progenitor/stem cells (n = 10) or bone marrow-derived mesenchymal stem cells (BM-MSCs; n = 6) and sacrificed at day 21 after PAN injection (Fig. 1B). Progenitor/stem cell treatment did not ameliorate kidney weight increase after PAN injection at 21 days and in all groups, kidney weight was higher in comparison to the normal kidney (Fig. 1C). Serum creatinine levels were lower in the c-kit treated group in comparison to the saline group at day 10 (**P = 0.0043), although no difference was observed in all three groups at day 21 (Fig. 1D). Similarly, blood urea nitrogen (BUN) levels were lower in the c-kit treated group when compared to the saline (***P = 0.0002) and MSC (*P = 0.037) treated groups at day 10, although no difference was observed in all three groups at day 21 (Fig. 1E). Rat weight was comparable in all three groups over time (S1A Supplementary Materials).

To examine the effect of stem cell treatment on podocyte injury after PAN injection, we assessed UACR in all three groups according to the time, using analysis of variances (Fig. 1F). No differences were found according to treatment. Next, we compared overall average of UACR by time-point as described by others^23^. At days 7, 10, 14 and 21, overall average of UACR was 0.37, 0.85, 0.45 and 0.31 g/creatinine (mg), respectively (Fig. 1G). At day 7, saline (~40%) and MSC (~67%)-treated groups exhibited higher UACR values when compared to normal kidneys (*P = 0.039 and **P = 0.0031, respectively), whilst c-kit treated group was not different from NK. These findings indicate that c-kit cell treatment attenuated the initial peak of PAN-induced albuminuria. At day 10, there was no difference between treated groups and normal kidneys. With the progression of PAN-mediated damage to kidneys, saline-treated group (~60%) exhibited higher UACR values when compared to normal kidneys (****P < 0.0001), MSC-treated group (~17%; *P = 0.038) and c-kit treated group (0%; ***P = 0.0002) at day 14. Lately, when PAN-mediated damage began spontaneous recovery at day 21, consistent with the self-limited nature of the model, we observed that saline treated group (~33%) maintained higher UACR values when compared to normal kidneys (*P = 0.04), but lower than MSC-treated group (~80%; *P = 0.018). C-kit treated group (10%) exhibited higher UACR values when compared to normal kidneys (****P < 0.0001), but lower UACR values when compared to MSC-treated group (***P = 0.0004).

Semi-quantitative injury scores were calculated for acute tubular necrosis, tubular casts, interstitial inflammation and mesangial expansion (Fig. 1H,I). Acute tubular necrosis score was the only score that was lower in the c-kit treated group when compared to the saline group at day 10 (Fig. 1H). This difference can help explain the lower levels of creatinine and BUN in the former group at this time-point. At day 21, injury scores were not different in all three groups (Fig. 1I). In addition, when data from all variables were pooled to obtain an overall injury score, no difference was observed according to the treatment and time (S1B Supplementary Materials). Of importance, we did not observe progression to focal segmental glomerulosclerosis.

**Figure 2:**
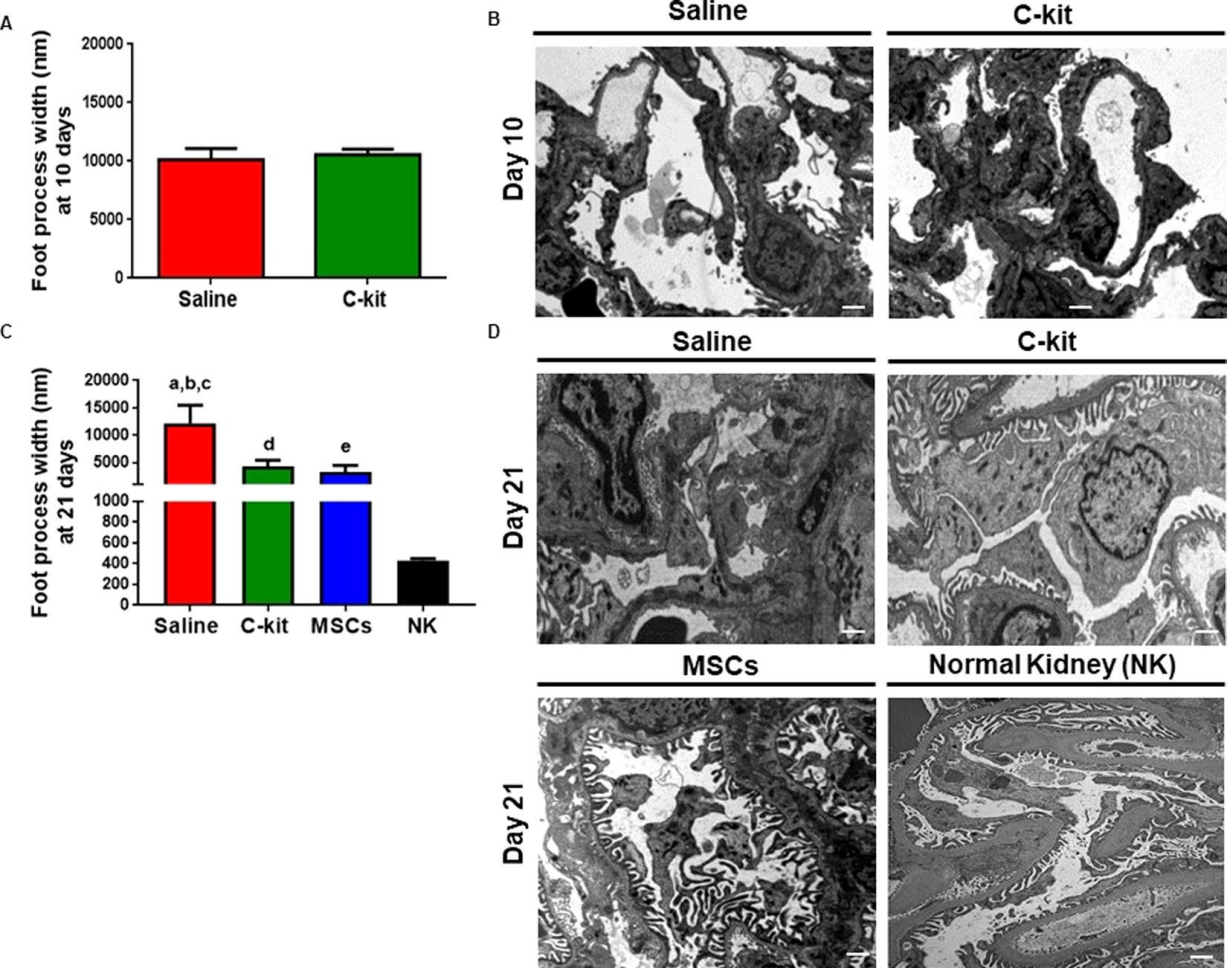
Progenitor/stem cell therapy restores foot process effacement. (**A**) Foot process width (FPW) was not different between saline and c-kit treated groups at day 10. (**B**) Representative images of the transmission electron microscopy (TEM) for saline and c-kit treated animals at day 10 (n = 4 in each group). (**C**) FPW was lower in the c-kit (a, *P = 0.03) and MSC (b, *P = 0.047) treated groups when compared to the saline group at day 21 (n = 4 in each group). FPW was higher in the saline, c-kit and MSC-treated groups when compared to normal kidneys (c,d,e; all ****P < 0.0001, respectively). (**D**) Representative images of the transmission electron microscopy (TEM) for saline, c-kit, and MSC-treated animals at day 21 (n = 4 in each group) and normal kidney (NK, n = 3). All data are means ± SEM. Scale bars represent 2 μm for (**B**) and 1 μm for (**D**).

Next, we assessed ultra-structural changes at days 10 and 21 and examined the effect of stem cell treatment on foot process effacement, the stereotypic reaction of podocyte to damage Foot process width (FPW) was measured as previously described^29^ and was comparable in the saline and c-kit treated groups at day 10 (Fig. 2A,B). However, FPW was significantly lower in the kidney-derived c-kit^+^ progenitor/stem cell and MSC-treated groups compared to the saline group at day 21 (Fig. 2B; *P = 0.03 and *P = 0.047, respectively), highlighting an important aspect of progenitor/stem cell therapy for glomerular disease (Fig. 2C,D). In saline treated group at day 21, FPW was also significantly higher when compared to normal kidneys (male SD rats 3–4 months-old; ****P < 0.0001) (Fig. 2C,D). Likewise, in those groups treated with progenitor/stem cell therapy at day 21, FPW was higher when compared to healthy controls (****P < 0.0001), indicating that these treatments promoted partial reversal of the injury (Fig. 2C,D).

### Kidney-derived c-kit^+^ progenitor/stem cells exhibit multi-compartment engraftment into the kidneys

**Figure 3:**
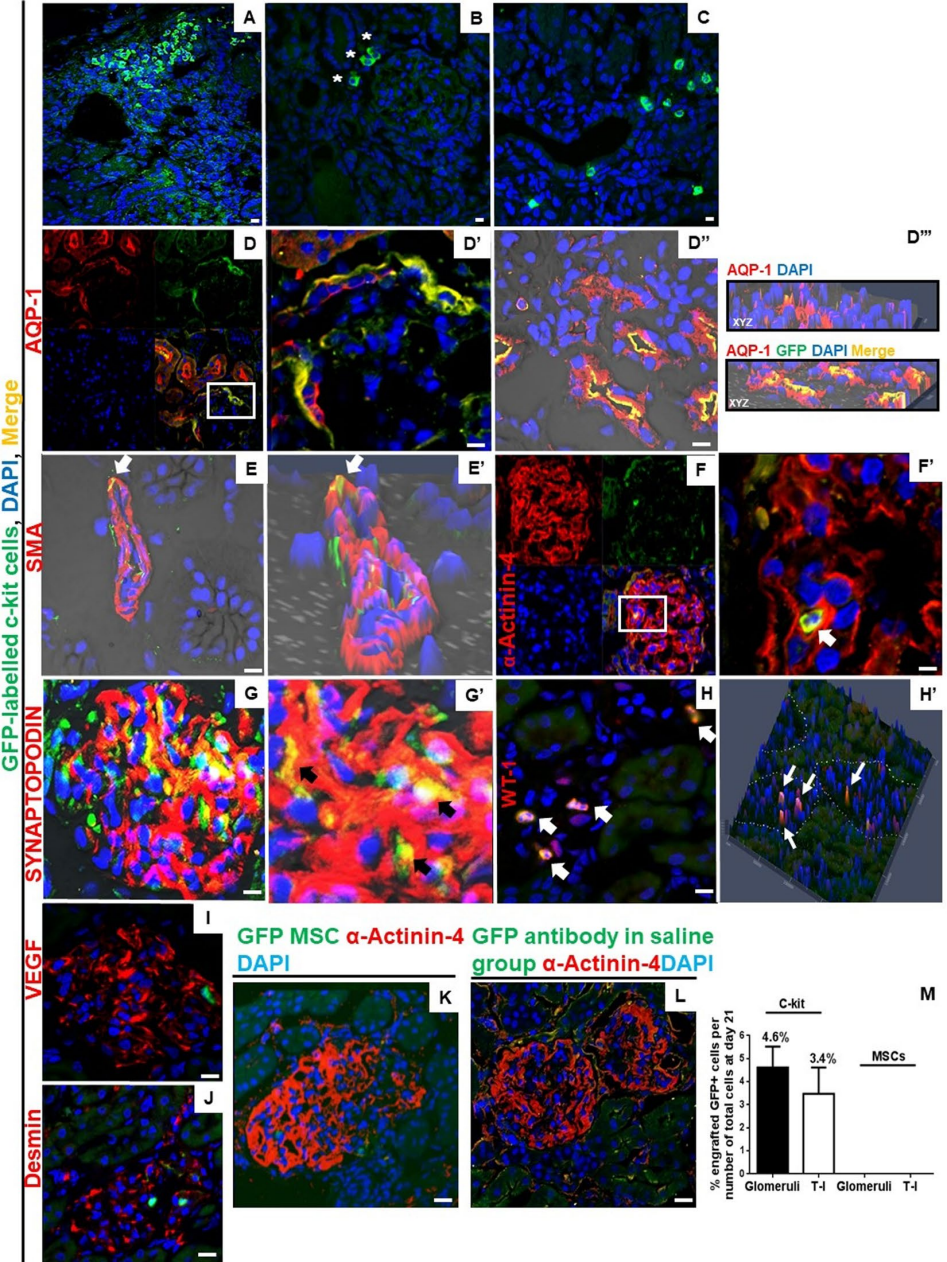
Kidney-derived c-kit^+^ progenitor/stem cells present multi-compartment engraftment and differentiate into podocyte-like cells after PAN injection. (**A**–**C**) GFP-labelled c-kit^+^ cells were observed in the interstitial compartment of cortex, including the peri-glomerular region (*), and in tubular compartment of cortex and corticomedullar regions. (**D**) GFP-labelled c-kit^+^ cells stained for aquaporin-1 (AQP-1) in the Bowman’s capsule. Inset shows region at higher magnification (**D’**). GFP-labelled c-kit^+^ cells also stained for AQP-1 in proximal tubules (**D”**). Orthogonal slides of three-dimensional reconstructed images at all three different planes allows visualization of coexpression of fluorescence signals AQP-1 (red), GFP (green), ensuring that the yellow signal is not due to cell stacking (**D”’**). (**E**) GFP-labelled c-kit^+^ cells stained for smooth muscle actin (SMA) in a vessel, as demonstrated by 2-D confocal image (**E’**) (arrows). (**F**) GFP-labeled c-kit^+^ cells stained for α-Actinin-4 in glomeruli. Inset shows region at higher magnification (**F’**) (arrow). (**G**) GFP-labeled c-kit^+^ cells stained for synaptopodin in glomeruli. Inset shows higher magnification (**G”**) (arrows). (**H**) GFP-labeled c-kit^+^ cells stained for WT-1 in a few podocytes. 3-D confocal image shows the co-localization (**H’**) (arrows). (**I**,**J**) Clusters of GFP-labeled c-kit^+^ cells were also found in glomeruli and did not stain for VEGF and desmin. (**K**) GFP-MSCs did not engraft at day 21 after PAN injection. (**L**) GFP-antibody in saline group. (**M**) On day 21, the number of GFP-labeled c-kit^+^ cells was 4.6 ± 0.91 and 3.4 ± 1.15% in the glomerular and T-I compartments, respectively. No engraftment was observed in the MSC treated group. Scale bars represent 20 μm for confocal images. At day 10, n = 5 and n = 8 for saline and c-kit-treated group, respectively, and n = 12, n = 10, and n = 6 for saline, c-kit, and MSC-treated groups at day 21, respectively.

To further substantiate the regenerative capacity of the kidney-derived c-kit^+^ progenitor/stem cells, we assessed the engraftment potential of these cells. Immunofluorescence staining with an anti-GFP antibody indicated that GFP-labelled c-kit^+^ cells were integrated into tubule-interstitial (T-I) compartment of both cortex and corticomedullar regions (Fig. 3A–C) in all c-kit-treated animals. Most of GFP-labelled c-kit^+^ cells engrafted into glomeruli were found in Bowman’s capsule, as demonstrated by the co-localization with aquaporin-1 (AQP-1) in the urinary pole (Fig. 3D-D’). GFP-labelled c-kit^+^ cells also stained for AQP-1 in proximal tubules (D”) and for smooth muscle actin in vessels (Fig. E-E’). A few GFP-labelled c-kit cells stained for podocyte markers, as documented by the co-localization with α-Actinin-4 (Fig. 3F-F’) and synaptopodin (Fig. G-G’). A few GFP-labelled c-kit^+^ cells also differentiated into podocyte-like cells and co-stained for WT-1 (Fig. H-H’; S2A Supplementary Materials). To note, GFP-labelled c-kit^+^ cells were observed in clusters inside the glomeruli and did not stain for desmin and VEGF (Fig. 3I,J). In our previous study^21^, we documented that c-kit sorted cells expressed renal markers, including WT-1, AQP-1, NKCC2, and NCCT, but not α-Actinin-4. One explanation for WT-1 detection in c-kit^+^ cells could be the fact that c-kit cells may represent a cellular kidney subfraction that can be induced to return to cap mesenchyma-like structures upon isolation.

On the other hand, no engraftment was observed in the GFP-MSC treated group (Fig. 3K). Anti-GFP antibody in saline sections was used as a control (Fig. 3L). On day 21, the number of GFP-labelled c-kit^+^ cells was 4.6 ± 0.91% and 3.4 ± 1.15% in the glomerular and T-I compartments, respectively, of all cells counted in 20x fields with 2x zoom (~4600 kidney cells counted, including ~1300 glomerular cells) (Fig. 3M). We found an average of 7.53 ± 6.6% GFP^+^ cells per glomeruli. From all counted glomeruli per animal (36.8 ± 1.9), 11.7 ± 9.8% (range, 2.8–22.2%) glomeruli exhibited GFP+ cell engraftment. Based on these findings, it is possible that glomeruli with cell engraftment improved, and those without engraftment remained injured.

### Kidney-derived c-kit^+^ progenitor/stem cells targeted podocyte cytoskeleton by modulating α-Actinin-4, but did not increase the number of podocytes nor induce the production of cytokines in the acute model of PAN-induced proteinuria

**Figure 4:**
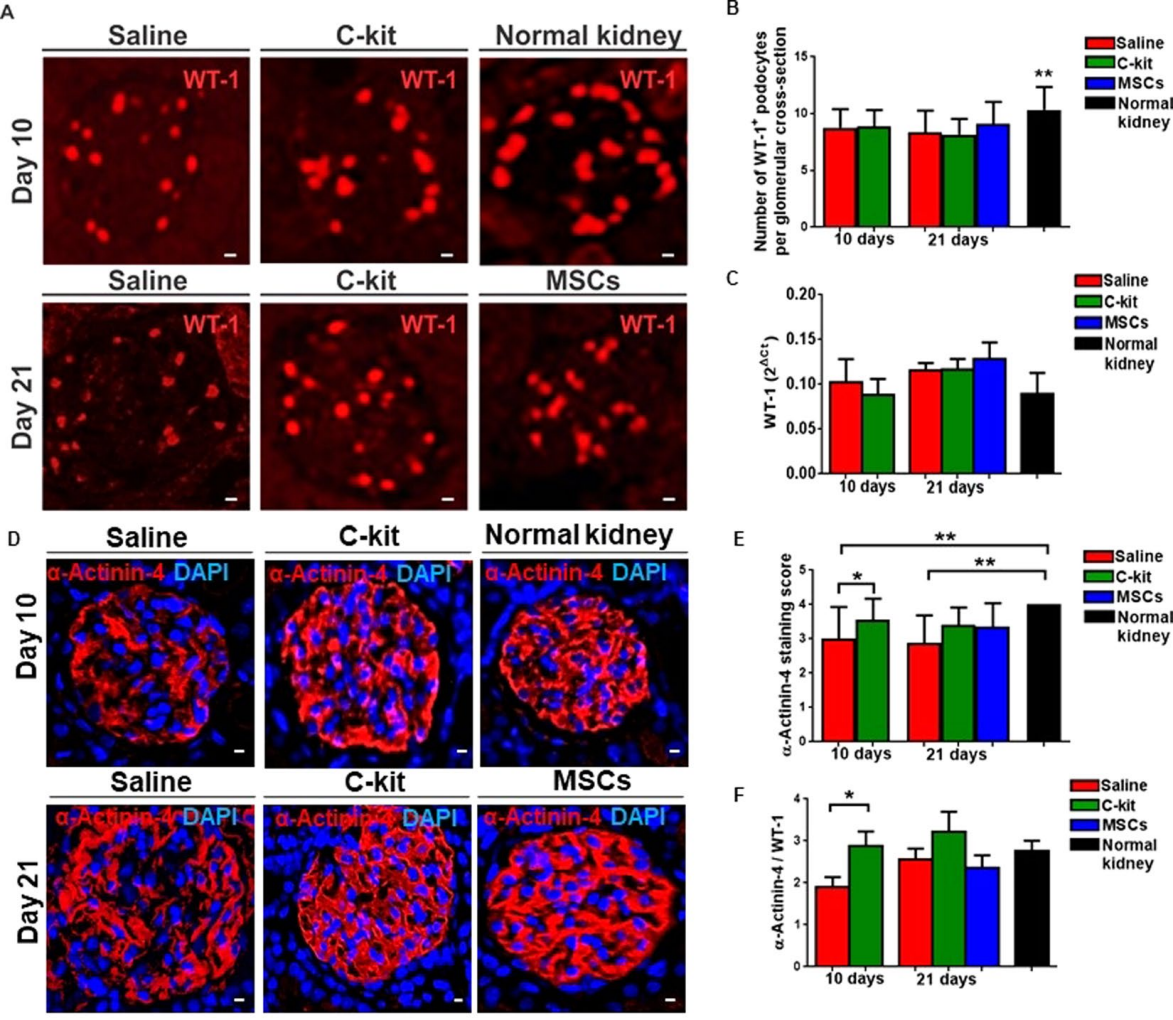
Kidney-derived c-kit^+^ progenitor/stem cells promote podocyte recovery by up regulating α-Actinin-4 involved in the podocyte cytoskeleton maintenance, but not by increasing the number of podocytes. (**A**) Number of podocytes verified by the immunofluorescence staining for WT-1. (**B**) Normal kidney exhibited higher number of WT-1^+^ podocytes when compared to all three groups injected with PAN (**P = 0.0017). (**C**) WT-1 gene expression was not different among groups, by qPCR (2^ΔCt^). (**D**) Immunofluorescence staining for α-Actinin-4 and DAPI. (**E**) Semi-quantitative analyses of α-Actnin-4 immunofluorescence staining score in all groups compared to normal kidney. C-kit treated group at day 10 score was significantly higher than saline group at day 10 (*P = 0.029). Saline group score was lower than normal kidney independently of the time (**P = 0.0084 and **P = 0.003 at days 10 and 21, respectively). (**F**) Alpha-Actnin-4 gene expression was normalized to WT-1 and was up regulated in the c-kit treated animals when compared to the saline group at day 10 (*P = 0.048), by qPCR (2^ΔCt^). Error bars represent means ± SEM. Scale bar represents 20 μm for confocal images in (A, D). At day 10, n = 5 and n = 8 for saline and c-kit-treated group, respectively, and n = 12, n = 10, and n = 6 for saline, c-kit, and MSC-treated groups at day 21, respectively.

To test whether the beneficial outcome from kidney-derived c-kit^+^ progenitor/stem cells or MSCs was attributed to preservation of podocyte number, we verified WT-1 expression by immunofluorescence (Fig. 4A). A minimum of 40 glomeruli per animal were counted to assess the number of WT-1^+^ podocytes. Normal kidney exhibited higher number of WT-1^+^ podocytes when compared to all three groups treated with PAN, independently of the time (Fig. 4B, **P = 0.0017). By qPCR, WT-1 expression was not different among groups (Fig. 4C), as well as nephrin, podocin, and synaptopodin, and the adaptor molecule CD2AP (CD2-associated protein) (S3A and S4A Supplementary Materials). Podocalyxin was down regulated in the c-kit treated group at day 10 (*P < 0.05), but reached normal levels at day 21 (S3A Supplementary Materials).

To further substantiate the involvement of progenitor/stem cell treatment in podocyte cytoskeleton, we calculated the α-Actinin-4 staining score using semi-quantitative analyses (Fig. 4D,E). C-kit treated group at day 10 score was significantly higher than saline group at day 10 (*P = 0.029), whereas saline group at day 10 and at day 21 scores were lower than normal kidney (**P = 0.0084 and **P = 0.003, respectively) (Fig. 4E). By qPCR, α-Actinin-4 expression was higher in the c-kit treated group when compared to the saline treated group at day 10 (*P = 0.048; unpaired *t*-test), although this expression was up regulated in both groups at day 21 (Fig. 4F). These results suggest that c-kit progenitor/stem cell treatment can potentially target α-Actinin-4 and ultimately rescue podocyte disarrangement and accelerate FPE reversal.

**Figure 5:**
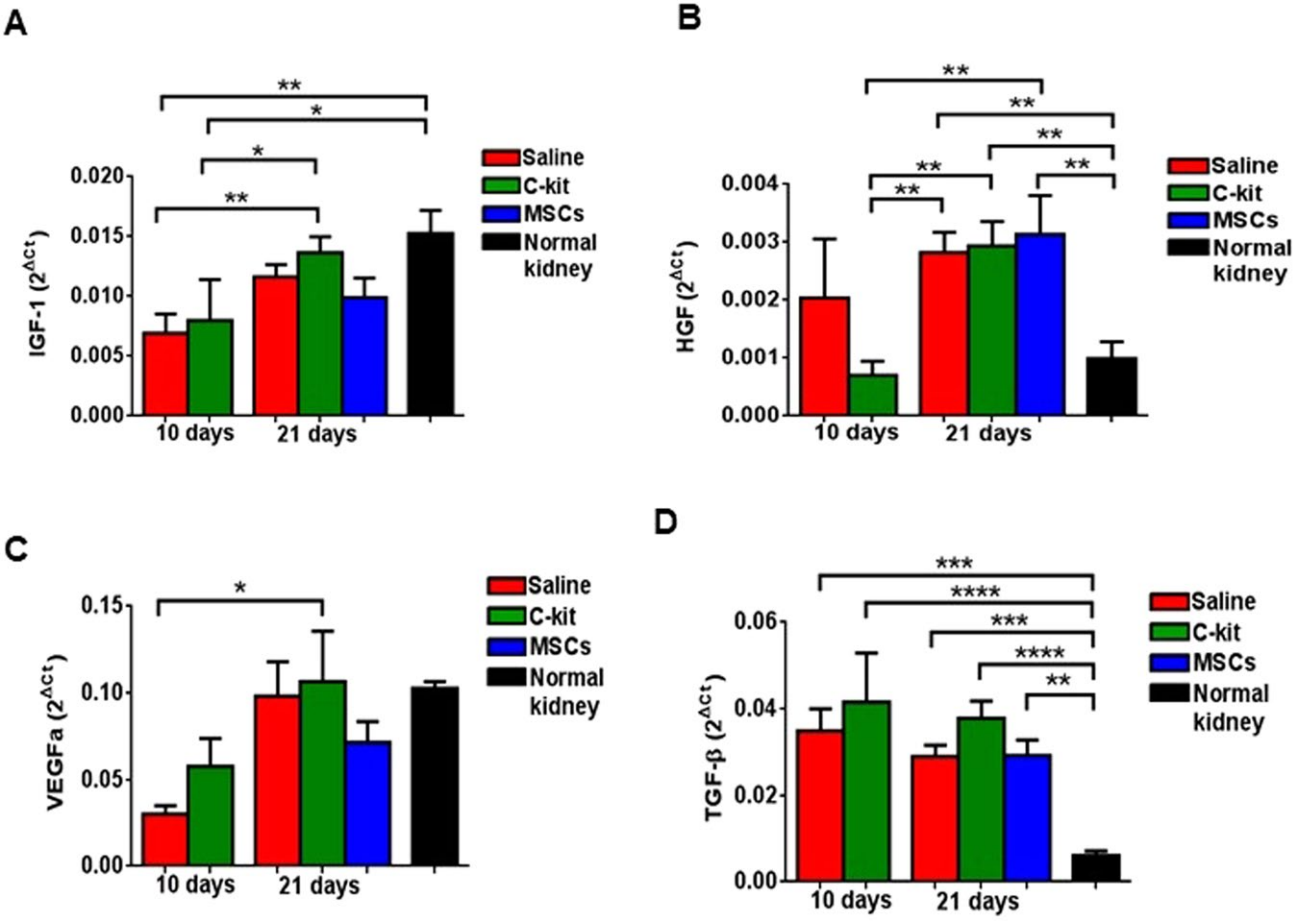
Kidney-derived c-kit^+^ progenitor/stem cells and BM-MSCs did not contribute to podocyte recovery by inducing the production of cytokines in the acute model of PAN-induced proteinuria. (**A**) Gene expression of insulin growth factor-1 (IGF-1) was not related to progenitor/stem cell treatment. (**B**) Hepatocyte growth factor (HGF) increased according to the time independently of the treatment as well. (**C**) Vascular endothelial growth factor A (VEGFa) was down regulated at day 10 in the c-kit and saline treated groups, but increased at day 21. (**D**) Transforming growth factor β (TGF-β) remained up regulated in all three groups when compared to the normal kidney. *P < 0.05; **P < 0.01; ***P < 0.001; ****P < 0.0001. Error bars represent means ± SEM, by qPCR (2^ΔCt^). At day 10, n = 5 and n = 8 for saline and c-kit-treated group, respectively, and n = 12, n = 10, and n = 6 for saline, c-kit, and MSC-treated groups at day 21, respectively.

To assess the paracrine effect of the kidney-derived c-kit^+^ progenitor/stem cells and MSCs, renoprotective growth factors, such as insulin growth factor-1 (IGF-1), hepatocyte growth factor (HGF), and vascular endothelial growth factor A (VEGFa) were measured and compared to saline-treated group and to normal kidneys (Fig. 5A–C). In addition, to test if cell-treated kidneys exhibited lower progression to fibrosis, we also measured the pro-fibrotic growth factor transforming growth factor-β (TGF-β) levels in kidney cortex by qPCR (Fig. 5D).

IGF-1 and HGF levels increased in the c-kit treated group in a time-dependent manner, but was not different from saline treated group. VEGFa did not play a significant role in this model as well. Likewise, in all groups, independently of the time, TGF-β levels were higher than the normal kidney. These findings support that cytokines were mainly produced by host kidney cells, and not by progenitor/stem cells, and that production was modulated when spontaneous proteinuria recovery occurred in the PAN model.

### Kidney-derived c-kit^+^ progenitor/stem cells accelerate podocyte recovery by activating autophagy

**Figure 6:**
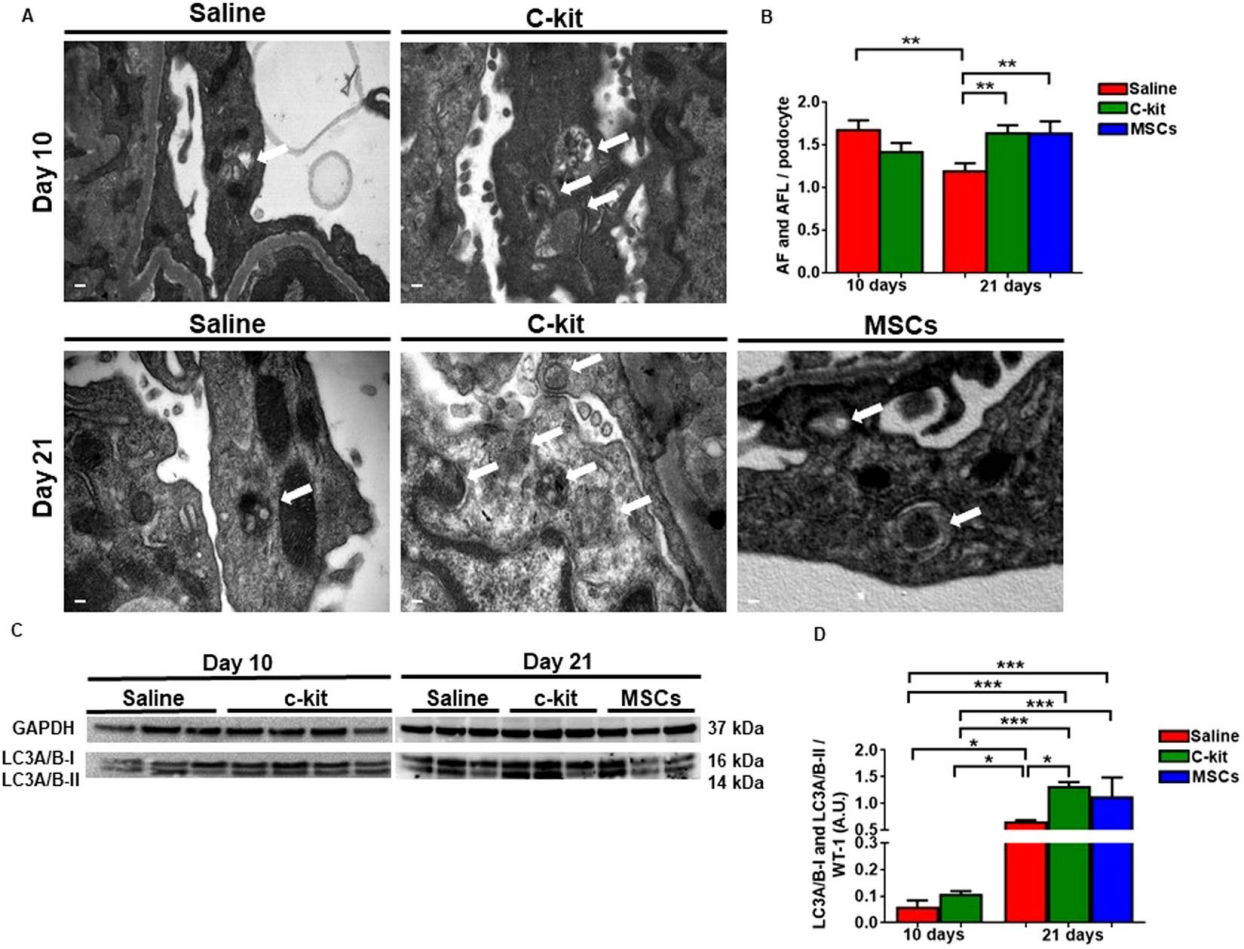
Kidney-derived c-kit^+^ progenitor/stem cells promote podocyte recovery by activating autophagy. (**A**) Representative TEM images of saline, c-kit and MSC treated groups according to the time. White arrows indicate autophagosomes (AF) and autophagolysosomes (AFL). (**B**) AF and AFL number was not different in the saline and c-kit-treated groups at day 10. At day 21, there was an increase in the number of AF and AFL in the c-kit and MSC-treated groups in comparison to the saline group (**P = 0.0013 and **P = 0.0067, respectively). In the saline treated group there was a decrease in the AF and AFL number according to the time (**P = 0.0044). (**C**) Representative Western blots of the microtubule-associated protein light chain 3 (LC3) A/B-I and LC3 A/B-II in all three groups according to the time. (**D**) LC3A/B-I and LC3A/B-II expression was higher in the c-kit treated group when compared to the saline group at day 21 (*P = 0.014; all normalized to WT-1), measured as arbitrary units (A.U.). *P < 0.05; **P < 0.01; ***P < 0.001. Error bars represent means ± SEM. Scale bar represents 0.2 μm for (A). N = 3 in saline group and n = 4 in c-kit group at day 10 and n = 3 in saline, c-kit and MSC-treated groups at day 21.

Next, we hypothesized that stem cell treatment might induce autophagy for the protection of podocyte against PAN-induced injury. Autophagy is an intracellular degradation system that maintains intracellular homeostasis by removing damaged proteins and organelles^30^. To test whether the beneficial outcome from kidney-derived c-kit^+^ progenitor/stem cells or MSCs may be attributed to the modulation of the autophagy, we counted the number of autophagosomes (AF) and the autophagolysosomes (AFL) per podocyte in 4600x or 5800x fields with 2x zoom (Fig. 6A). AF and AFL number was not different in the saline and c-kit-treated groups at day 10 (Fig. 6B). However, at day 21, there was an increase in the number of AF and AFL in the c-kit and MSC-treated groups in comparison to the saline group (Fig. 6B; **P = 0.0013 and **P = 0.0067, respectively). In the saline treated group there was a decrease in the AF and AFL number according to the time (**P = 0.0044).

Autophagy marker microtubule-associated protein light chain 3 (LC3) is used to assess autophagy activity and has two isoforms. LC3-I is converted to LC3II and then to autophagic vesicles^31^. We pursued further investigation of LC3A/B-I and II expression in podocytes by normalizing protein levels to WT-1 and found that autophagy increased with time after PAN injection and was more intense in the c-kit treated group when compared to the saline group at day 21 (Fig. 6C,D; *P = 0.014).

### Kidney-derived c-kit^+^ progenitor/stem cells modulate mTOR pathway and up regulate Rictor in podocytes

**Figure 7:**
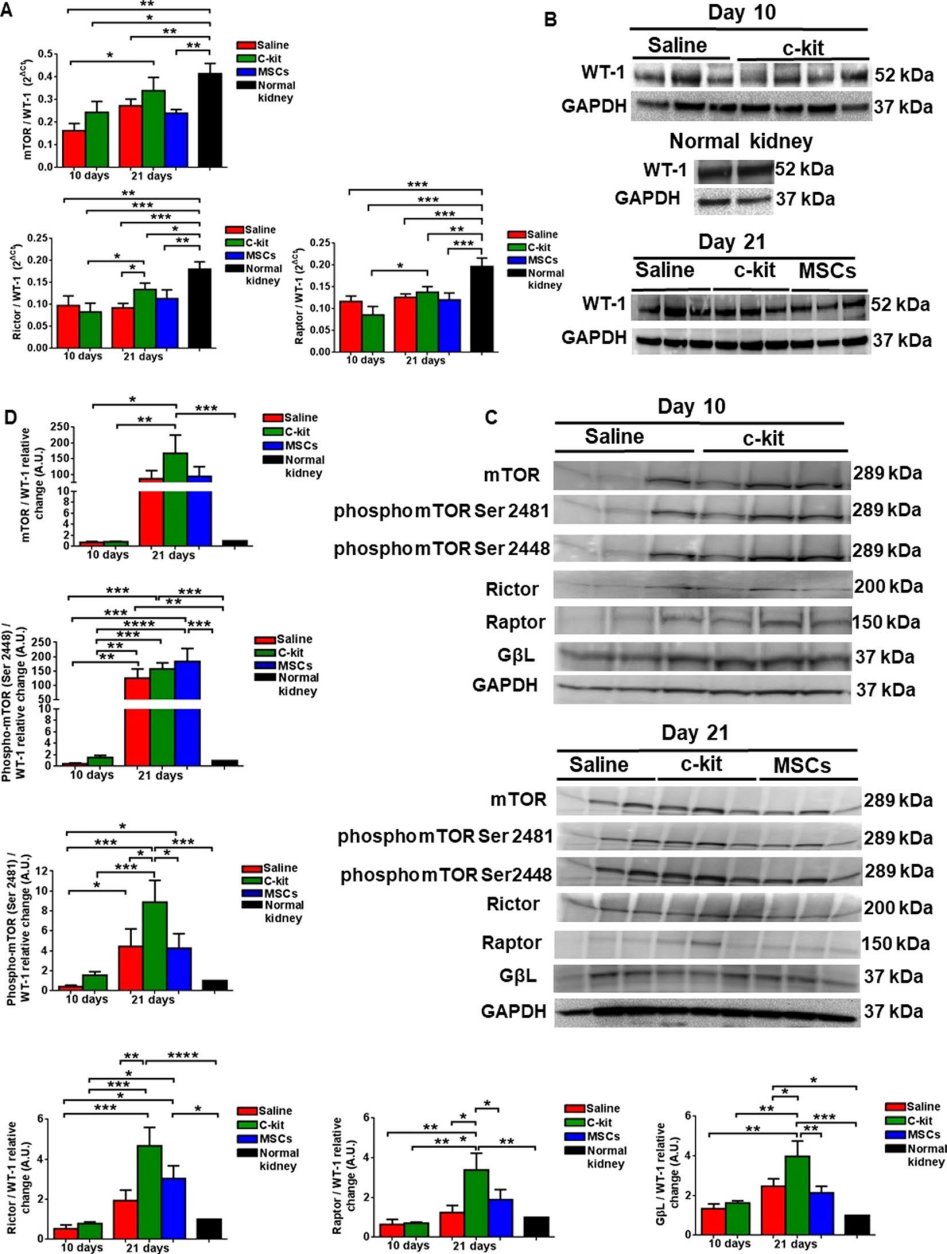
Kidney-derived c-kit^+^ progenitor/stem cells modulate mTOR pathway in a time- dependent manner and up regulate Rictor in podocytes. (**A**) qPCR analyses of mTOR pathway in podocytes calculated by normalizing the data to WT-1 levels. Rictor and Raptor levels were down regulated in all groups independently of the time when compared to the normal kidney, except mTOR expression in the c-kit treated group at day 21. Rictor expression was higher in the c-kit treated group in comparison to the saline group at day 21. (**B**) Representative Western blots showing the levels of WT-1 in kidney cortex according to the treatment and time in the saline, c-kit and MSC-treated groups and in normal kidney. (**C**) Representative Western blots of mTOR pathway according to the treatment and time in the saline, c-kit and MSC-treated groups. (**D**) mTOR, Rictor, Raptor, phospo mTOR 2448 and 2481, and GβL increased in the c-kit group from day 10 to day 21 (all normalized to WT-1). When compared to the saline group at day 21, c-kit treated group exhibited higher levels of Rictor and phospho mTOR 2481, and Raptor as well, but not phospho mTOR 2448. Comparison was done accordingly to the normal kidney (set as 1), measured as arbitrary units (A.U.). *P < 0.05; **P < 0.01; ***P < 0.001; ****P < 0.0001. Error bars represent means ± SEM. N = 3 in saline group and n = 4 in c-kit group at day 10 and n = 3 in saline, c-kit and MSC-treated groups at day 21.

The mammalian target of rapamycin (mTOR) is an evolutionarily conserved serine-threonine kinase that interacts with regulatory associated protein of mTOR (Raptor) or Raptor independent component of mTOR (Rictor) to form mTORC1 and mTORC2 complexes, respectively^32^. Next, in order to assess mTOR pathway in podocytes, we normalized mRNA levels of mTOR, Raptor and Rictor to WT-1 levels. The main findings included the down regulation of mTOR in all groups, except in the c-kit treated group at day 21, in comparison to the normal kidney (Fig. 7A). Rictor/WT-1 and Raptor/WT-1 levels were down regulated in all groups, independently of the time, when compared to the normal kidney. However, Rictor/WT-1 levels were higher in the c-kit treated group when compared to the saline treated group at day 21 (*P < 0.05). To note, Rictor/WT-1 and Raptor/WT-1 levels were up regulated in the c-kit treated group according to the time (*P < 0.05).

WT-1 expression at protein levels in kidney cortex was not different in all treated groups at days 10 and 21 when compared to the normal kidney by western blot analyses (Fig. 7B), supporting the immunofluorescence analyses (Fig. 4A). Next, we analyzed mTOR pathway signaling components (Fig. 7C) and normalized them to WT-1 expression, as previously described by others^33,34^. We found that some of these components were also up regulated in a time-dependent manner, notably Rictor and Raptor in the c-kit treated group compared to the saline group at day 21, by western blot analyses (Fig. 7D). To provide further evidence that mTOR was phosphorylated in a time-dependent manner, we documented that phospho mTOR Ser 2448, the main site of mTORC1 phosphorylation, was up regulated in the c-kit, MSC and saline treated groups at day 21 when compared to day 10 and to normal kidney. Conversely, phospho mTOR Ser 2481, the main site of mTORC2 phosphorylation, and GßL were mainly up regulated in the c-kit treated group when compared to the saline and MSC-treated groups at day 21 (Fig. 7D). In conclusion, c-kit treated group exhibited higher levels of Rictor and phospho mTOR 2481, and Raptor as well, but not phospho mTOR 2448, when compared to the saline group at day 21.

**Figure 8:**
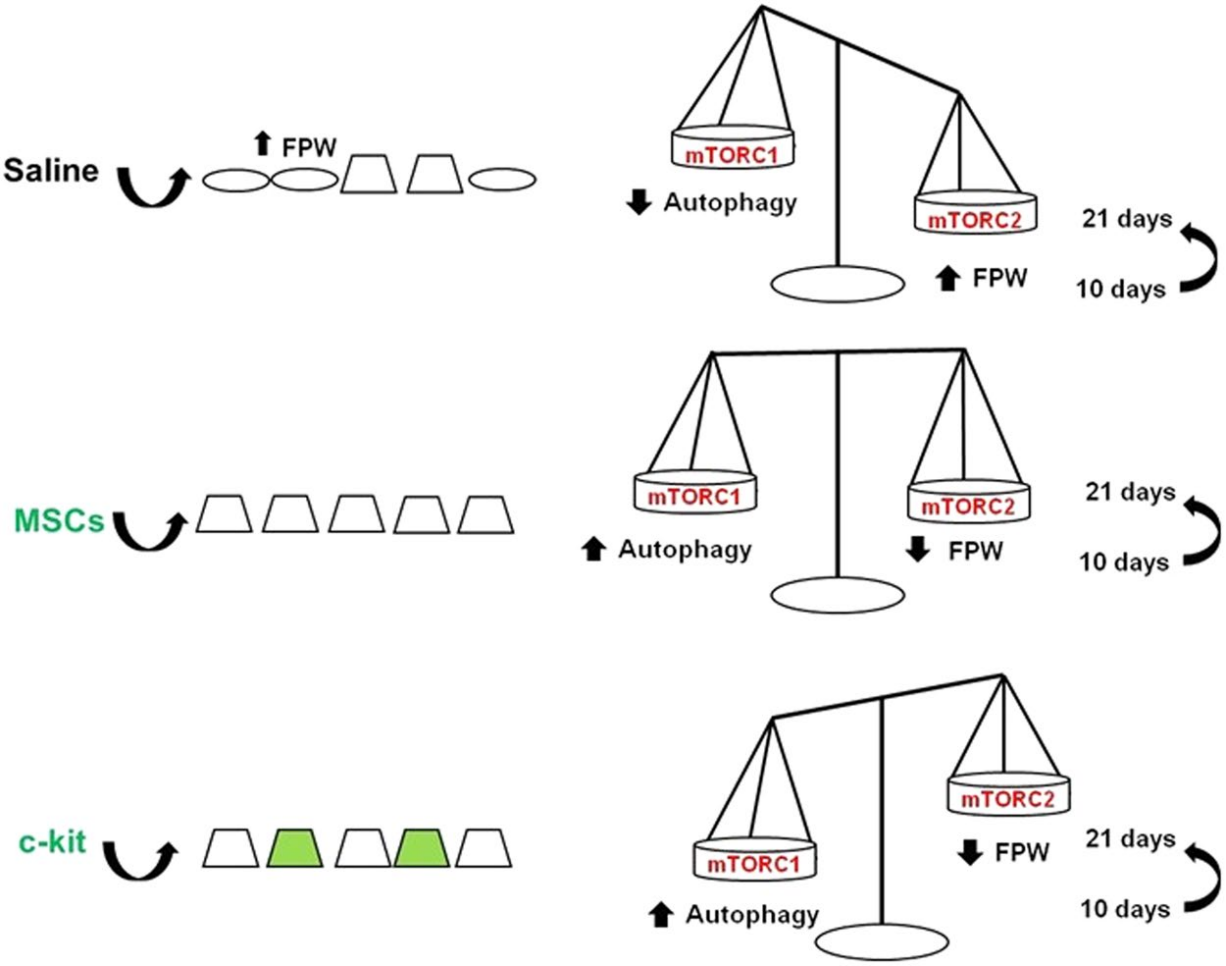
Proposed mechanisms of foot process width (FPW) improvement according to the treatment after PAN injection. Kidney-derived c-kit^+^ progenitor/stem cells and BM-MSC contributed equally to accelerate podocyte recovery in a time-dependent manner, although c-kit^+^ progenitor/stem cells exhibited multi-compartment engraftment and differentiated into podocyte-like cells. These cells activated autophagy and targeted podocyte cytoskeleton by modulating α-Actinin-4 rearrangement and mTORC2 pathway as well.

In Fig. 8, we summarized the main mechanisms involved in kidney recovery mediated by c-kit^+^ progenitor/stem cells and MSCs.

## Discussion

Our present findings suggest an important role of kidney-derived c-kit^+^ progenitor/stem cell treatment in the maintenance of the permeability barrier of the kidney in the model of acute proteinuria induced by PAN.

The dysregulation of the highly specialized podocyte actin cytoskeleton is closely associated with a disease phenotype^35^. With podocytes being terminally differentiated cells that do not proliferate, it is reasonable that regulation of actin cytoskeleton rearrangement in podocyte foot processes plays an important role in podocyte regeneration. Here we documented for the first time that kidney-derived c-kit^+^ progenitor/stem cell and MSC treatment targeted the actin cytoskeleton in podocytes and both accelerated the reversal of foot process effacement (FPE) after PAN injection, representing an important therapeutic approach for glomerular disease. While, we did not observe a significant difference in proteinuria in all three groups at day 21, there was less severe FPE in the stem cell treated groups suggesting improvement in podocyte injury when compared to saline injected animals. Although the relationship between FPE and proteinuria in glomerular disease is well established, there is a poor correlation of quantitative morphometric analysis and proteinuria levels in human glomerular diseases^36,37^. Moreover, PAN model is self-limited, yet proteinuria is transitory and spontaneous recovery is anticipated, which may at least explain the lack of difference in proteinuria and serum creatinine and BUN levels among groups at day 21. This model is characterized by little, if any glomerular changes on light microscopy, which explain at least in part the absence of difference in histological analysis among PAN-injected groups. Likewise, PAN model is not a robust model of proteinuria as a chronic model of kidney injury. Further studies are therefore required to assess the effect of cell therapy on podocyte damage in that setting. Of importance, PAN model possesses a dose-dependent effect of podocyte damage and severity. Thus, PAN doses of 15 mg are not associated with focal segmental glomerulosclerosis (FSGS)^38^. Our study is in line with these findings. Conversely, multiple injections of PAN during 5 months resulted in sustained severe proteinuria and FSGS^39^.

To note, higher levels of α-Actinin-4 or its redistribution in c-kit treated group at day 10 (Fig. 4B) were detected a couple of days after PAN-induced peak of proteinuria, and may precede podocyte cytoskeleton rearrangement in response to change in the microenvironment, and contribute ultimately to proteinuria reduction. Although progenitor/stem cell therapy improved α-Actinin-4 levels, further studies are required to analyze the impact of that treatment on intercellular adhesion molecules, such as ICAM-1 and -2, and ß1-integrins. Subnormal levels of these adhesion molecules after PAN injection may explain the increased proteinuria and the FPW when α-Actinin-4 levels are high, suggesting that the foot process architecture is not completely recovered yet. In line with these observations, recent data documented that treatment with ß1-integrin agonist prevented damage-induced decreases in F-actin stress fibers, as well as reduced peak proteinuria caused by PAN in rats^40^. Likewise, podocyte cytoskeletal disarrangement can be attributed to intracellular α-Actinin-4 redistribution due to injury. In diabetes, for example, high glucose and advanced glycosylation end products (AGE) induced the inner cytoplasmic re-localization of α-Actinin-4 and disrupted actin fibers^41^.

Of importance, higher levels of UACR in MSC-treated group can also be attributed to capillary clogging after cell injection due to its size^42^, to intra-glomerular differentiation to adipocytes^16^, to an inflammatory response after cell injection^43^, and to the lack of both MSC engraftment and MSC differentiation into kidney structures. For kidney-derived c-kit^+^ progenitor/stem cells, the mechanisms involved in podocyte recovery included mainly paracrine effects. Direct differentiation into kidney structures, such as podocyte, tubular, and vascular-like structures occurred in small quantity.

Although the number of WT-1^+^ cells was not higher in the c-kit treated group, in chronic models of injury, where podocyte damage is permanent, c-kit^+^ progenitor/stem cell engraftment may be more evident. Cell engraftment occurred mainly into Bowman’s capsule, as reported for the CD24^+^CD133^+^ renal progenitors^5^, suggesting that podocyte-like cells formed could have been recruited from parietal epithelial cells^6^, although in small quantity. Tubular damage can also occur after PAN injection^44,45^, but tubular and vascular c-kit cell engraftment and paracrine mechanisms could both explain the less severe ATN score in the c-kit treated group at day 10, as previously documented after acute ischemia-reperfusion injury^21^. However, c-kit cells may require interaction with other kidney cells, such as Lgr4^+^ cells^46,47^, to differentiate in those structures. Furthermore, a limitation of our study is that lineage tracing studies are required to document c-kit cell fate during kidney development and in different models of kidney injury.

Progenitor/stem cell delivery route is also a crucial aspect of cell therapy. In intravenous route, the number of cells, multiple intravenous injections and cell size increase the chance of pulmonary trapping^42,48^. Although intra-parenchymal administration of progenitor/stem cells also has beneficial effect on kidney repair, this route is less practical for clinical application, especially when renal disease is diffuse. Conversely, arterial route for progenitor/stem cell delivery promote kidney regeneration more efficiently than intravenous route, as assessed in a recent meta-analysis of MSC treatment in kidney injury^49^. In line with those observations, we believe that intra-arterial route contributed to c-kit^+^ cell engraftment to the paracrine effect of both c-kit^+^ cells and MSCs.

Of importance, stem cell therapy may lead to either kidney functional or morphological impairments, such as a transitory increase in serum creatinine^50^, stem cell maldifferentiation accompanied by glomerulosclerosis^16^, hyperplastic lesions^10^, appearance of angiomyeloproliferative lesions^17^, and low rates of cell engraftment as well^18^. However, the results obtained from these studies will collectively set the basis for establishing further investigation on the therapeutic potential of progenitor/stem cells for treatment of kidney disease in preclinical and clinical studies, yet the benefit of progenitor/stem cell therapy outweighs the risk of therapeutic infusion.

To isolate and expand *ex-vivo* kidney-derived c-kit^+^ progenitor/stem cells from humans will be challenging, yet their spatiotemporal distribution during homeostasis and injury requires further studies on lineage tracing. In addition, ethical aspects are involved in the isolation of these cells from embryonic and neonatal tissues. Therefore, the search for allogeneic kidney-derived c-kit^+^ progenitor/stem cells obtained from deceased donors and the development of inducible pluripotent stem cells need to be widely pursued.

Our data support that α-Actinin-4 up regulation was associated with lower FPW measurement and could be thereafter used as a marker of podocyte cytoskeleton maintenance. At earlier time-points after PAN injection, α-Actinin-4 induction was demonstrated to precede FPE^51^, although others did not document that correlation^52^. Furthermore, low α-Actinin-4 levels were associated with progression of glomerulopathy and proteinuria in human diabetic nephropathy^53^. Of note, α-Actinin-4 is crucial for actin rearrangement after podocyte injury^28,54,55^ and normal podocyte adhesion^56^. The importance of the actin cytoskeleton in glomerular and podocyte function is also highlighted by mutations in α-Actinin-4, which leads to familial FSGS^57^ and by the severe glomerular disease in α-Actinin-4 deficient mice^58^.

Although we did not evaluate glomerular volume, it was documented that decreased glomerular volume may have a protective effect on the podocytes, preventing them from detaching, and thereby hindering the development of FSGS^38,59^. Thus, decreased glomerular volume in the course of PAN-induced injury may explain at least in part the improvement in functional parameters whilst podocyte cytoskeleton reorganization is still occurring.

Paradoxically, transitory down regulation of podocalyxin (S3A Supplementary Materials) may correspond to changes in podocyte cytoskeleton reorganization^60^ or be related to the expression in other cells, such as endothelial cells^61^.

Since podocytes have limited capacity to regenerate, the pro-survival mechanisms are critically important to maintain their viability. IGF-I^62,63^, VEGFa^64^, HGF^65–67^ contribute to maintenance of podocyte cytoskeleton by decreasing apoptosis and inflammation. Of importance, VEGFa is also produced by kidney-derived c-kit^+^ progenitor/stem cells^21^ and BM-MSC^11,68^, however local production by podocytes also contributed for maintenance of glomerular filtration barrier, notably for its action in the endothelial glomerular compartment^69^. Likewise, surviving cells may also have contributed to the production of cytokines (IGF-1, VEGFa, and HGF) and therefore to tissue repair, because their levels were comparable to the progenitor/stem cell treatment at day 21. Accordingly, injected c-kit cells and MSCs may modulate host kidney cells to secrete those growth factors, a mechanism that also contributed to our findings.

TGF-β is a pleiotropic cytokine implicated in pathogenesis of renal fibrosis and, ultimately, end-stage kidney diseases^70–72^. Although high levels of TGF-β were detected in all groups, independently of the time and treatment, renal fibrosis was not observed in a follow-up of 3 weeks after PAN injection. Longer follow-ups or chronic models of glomerular injury can provide a definitive conclusion about the impact of progenitor/stem cell treatment on TGF-β levels.

Podocytes exhibit higher levels of autophagy as a key homeostatic mechanism to maintain their integrity^23^. In agreement with these data, stimulation of autophagy by kidney-derived c-kit^+^ progenitor/stem cells and MSCs unravels an important renoprotective aspect of cell therapy. Furthermore, in other cells, such as the human placental MSCs, stem cell factor/c-kit pathway is involved in the balance of cell survival and death events by modulating autophagy^73^. An inverse correlation between podocyte recovery from PAN-induced nephrosis and LC3, a microtubule-associated protein, in podocytes explain the role of autophagic pathway in the reorganization of foot processes in podocytes^24^. Moreover, the amount of LC3-II in podocytes could be a good indicator of podocyte damage and therefore may represent a diagnostic tool. Some predicted consequences of the decline in autophagy are the inefficient clearance of damaged components and a poor response to stress^30^. To note, autophagy activation is correlated to mTORC1 inhibition in podocytes^74^ and kidney tubules^75^. Here we also documented that progenitor/stem cell therapy was associated with mTORC1 down regulation, which promoted activation of autophagy in a time-dependent manner. mTORC1 modulation correlated to phospho mTOR Ser 2448 levels, which represents its main site of phosphorylation^76^. Of note, mTORC1 activation may be attributed to the growth factor IGF-1 and to the oxidative stress, the hallmark of PAN-induced injury^77^.

Disruption of actin cytoskeleton induced by PAN or Heymann nephritis can be rescued by different pharmaceutical agents^78,79^, including mTOR inhibitors^80,81^. Tight balance of mTOR activity is therefore important for podocyte homeostasis maintenance. Podocyte mTORC1 deletion induces proteinuria and progressive glomerulosclerosis^26^. Paradoxically, increased mTOR activity can lead to early glomerular hypertrophy and hyperfiltration in diabetic nephropathy and when this activity is inhibited, progression of glomerular disease is ameliorated^26^. mTORC1 and downstream targets p70S6k and 4E-BP are mainly responsible for cell growth and proliferation in response to growth factors, nutrients, or stress^82^. mTORC2 and downstream targets AKT, PKC, and SGK1^83^ regulate actin cytoskeleton^84,85^. Our data suggest that Rictor up regulation may have contributed to FPE reversal after progenitor/stem cell treatment. Of note, mTORC2-AKT2-Rac1 represents a novel stress-responsive axis involved in podocyte adaptation to nephron loss and retardation of chronic kidney disease^86^, a mechanism that includes phospho mTOR Ser 2481 modulation, which represents the main site of mTORC2 phosphorylation^76^. Germline deletion of Rictor in mice causes embryonic death^87^, whereas tissue-specific deletion of Rictor often results in only minor phenotypes in skeletal muscle^88^, adipose tissue^89^ or kidney^26^. Importantly, none of those conditional knockout mice exhibited changes in the actin organization, whereas double knockout of mTORC1 and mTORC2 aggravated the phenotypes in kidney^26,86^, but not in skeletal muscle^88^. In addition, Rictor deletion in brain or Purkinje cells affects size and neuron morphology^90^.

## Conclusions

Here, our study documented that kidney-derived c-kit^+^ cells have renoprotective effects in a model of acute proteinuria induced by PAN in rats. These cells contributed to reversal of FPE by activating autophagy, as demonstrated by the number of autophagosomes and autophagolysosomes, and targeting podocyte cytoskeleton through α-Actinin-4 modulation. Moreover, c-kit progenitor/stem cell treatment modulated mTOR pathway in a time-dependent manner and activation of mTORC2-phospho mTOR Ser 2481 may explain at least in part the reorganization of podocyte cytoskeleton after PAN injection. Likewise, kidney-derived c-kit^+^ cells engrafted into the kidneys in very low percentage and differentiated occasionally into podocyte, vascular and tubular-like cells. Although this mechanism of differentiation seems to be inefficient to promote kidney repair, it occurred and, therefore, brings to the light putative direct benefit of c-kit^+^ cells in association with paracrine cell effect. Taken together, kidney-derived c-kit^+^ progenitor/stem cells may have the potential to treat kidney diseases characterized by podocyte damage.

## Electronic supplementary material

Supplementary Materials
