# Supplementary material for: Kidney-derived c-kit+ progenitor/stem cells contribute to podocyte recovery in a model of acute proteinuria
Source: Sci Rep. 2018 Oct 3;8:14723. doi: 10.1038/s41598-018-33082-x (PMC6170432; doi:10.1038/s41598-018-33082-x)
Supplement: Supplementary file 1 — Supplementary Materials [file 41598_2018_33082_MOESM1_ESM.pdf]

Supplementary Materials

Kidney-derived c-kit$^+$ progenitor/stem cells contribute to podocyte recovery in a model of acute proteinuria

Running title: Podocyte recovery by c-kit cells

Erika B Rangel[1,2,3], Samirah A Gomes[1,4], Rosemeire Kanashiro-Takeuchi[1,5], Russell G Saltzman[1], Changli Wei[6], Phillip Ruiz[7], Jochen Reiser[6], Joshua M Hare[1,5,8]

1 Interdisciplinary Stem Cell Institute, Leonard M Miller School of Medicine University of Miami, Miami, 33136, Florida, USA

2 Sociedade Beneficente Albert Einstein, Albert Einstein Hospital, São Paulo, 05652, São Paulo, Brazil

3 Federal University of São Paulo, São Paulo, 04023, São Paulo, Brazil

4 Laboratory of Cellular, Genetic, and Molecular Nephrology, Renal Division, University of São Paulo, 01246, São Paulo, Brazil

5 Department of Molecular and Cellular Pharmacology, Leonard M Miller School of Medicine, University of Miami, Miami, 33136, Florida, USA

6 Department of Medicine, Rush University Medical Center, Chicago, 60612, Illinois, USA

7 Departments of Surgery and Pathology, Leonard M Miller School of Medicine University of Miami, Miami, 33136, Florida, USA

8 Division of Cardiology, Leonard M Miller School of Medicine University of Miami, Miami, 33136, Florida, USA

Supplementary Figures

Figure 1. Functional and morphological changes in the saline, c-kit, and MSC treated groups according to the time.

(A) Rat weight (g) according to the treatment and time.

(B) Overall injury score according to the treatment and time.

Error bars represent means ± SEM.

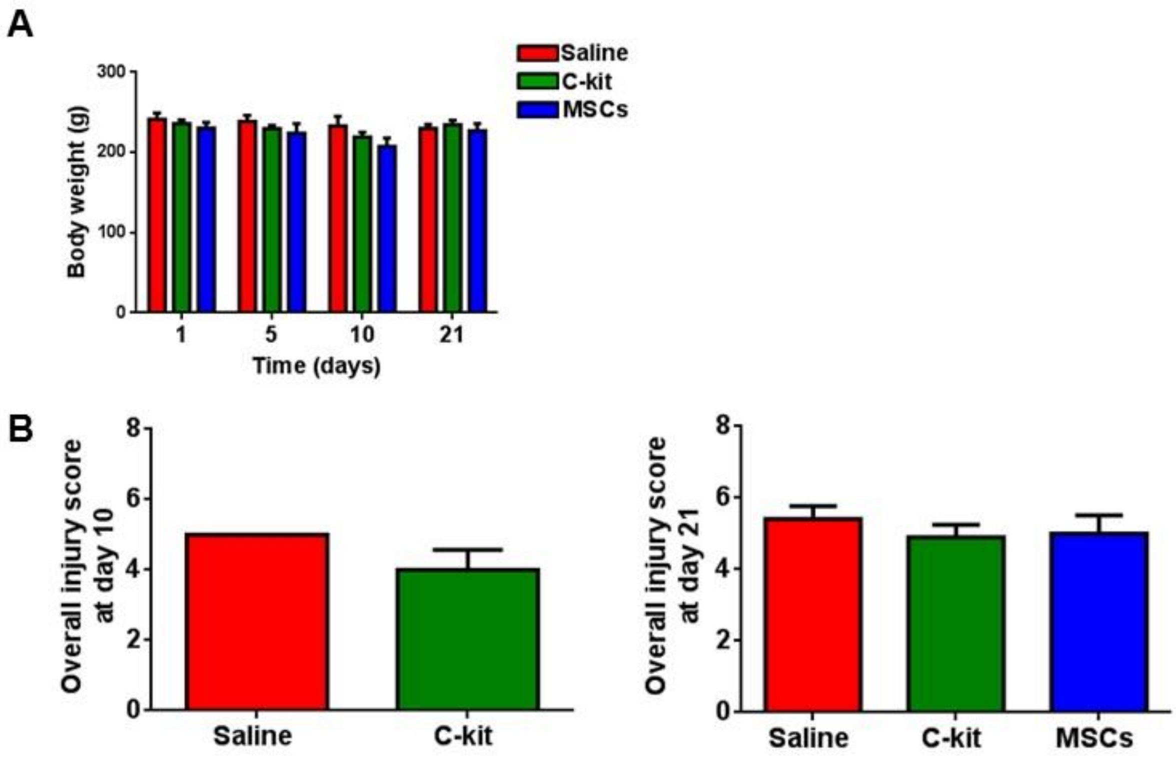

Figure 2. GFP-labeled c-kit⁺ cells stained for WT-1 in a few podocytes.

(A) Confocal image shows the co-localization of GFP-labeled c-kit$^+$ cells and WT-1, although WT-1 positive cells that did not stain for GPF (arrowheads) were also found. Scale bars represent 20 μm.

A

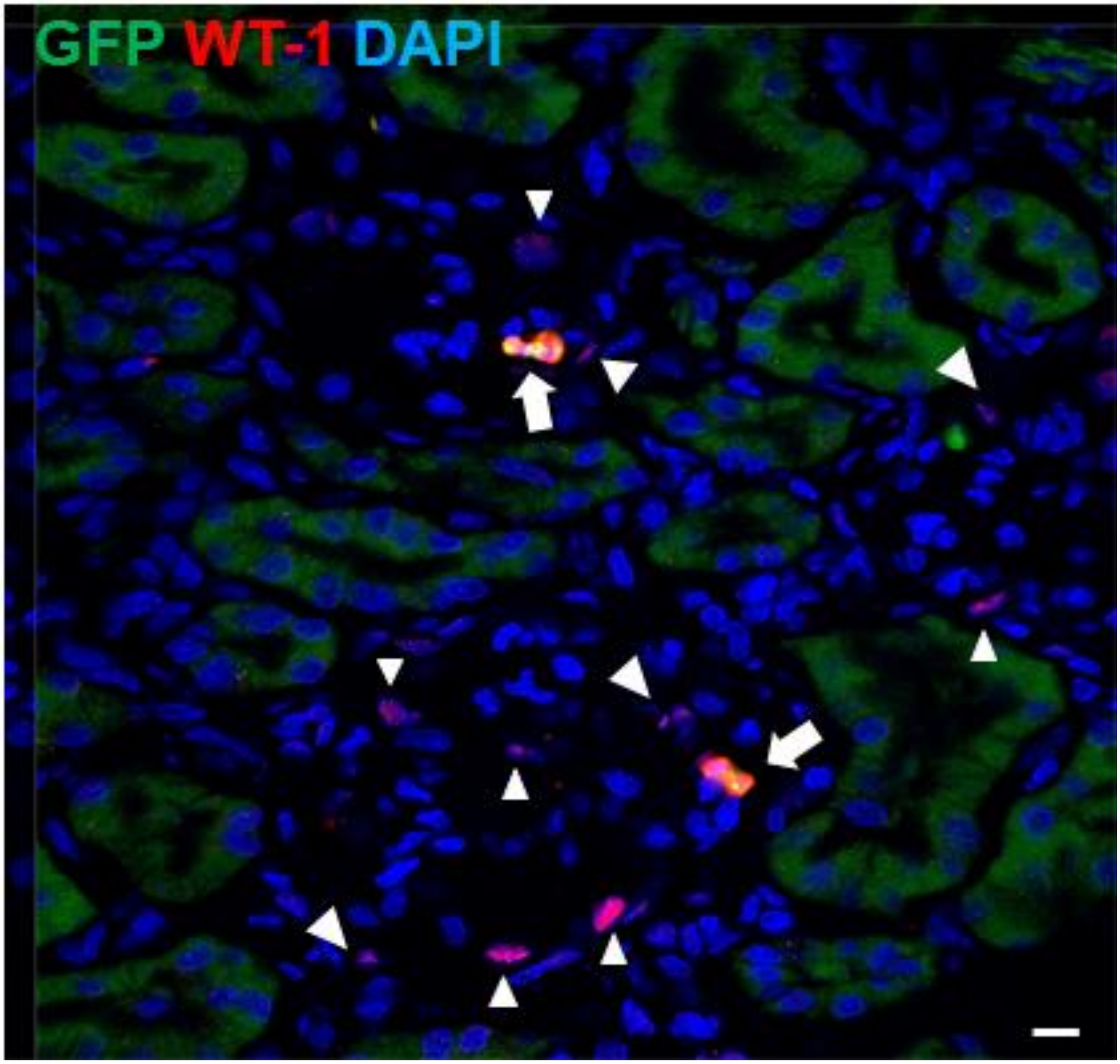

Figure 3. Gene expression after saline, c-kit$^+$ progenitor/stem cell and MSCs treatment.

(A) Gene expression of nephrin, podocin, synaptopodin, podocalyxin (PDX), CD2-associated protein (CD2AP) and α-Actinin-4 in kidney cortex in all three groups according to the time and compared to the normal kidney, by qPCR ($2^{\Delta Ct}$).

**P*<0.05. Error bars represent means ± SEM.

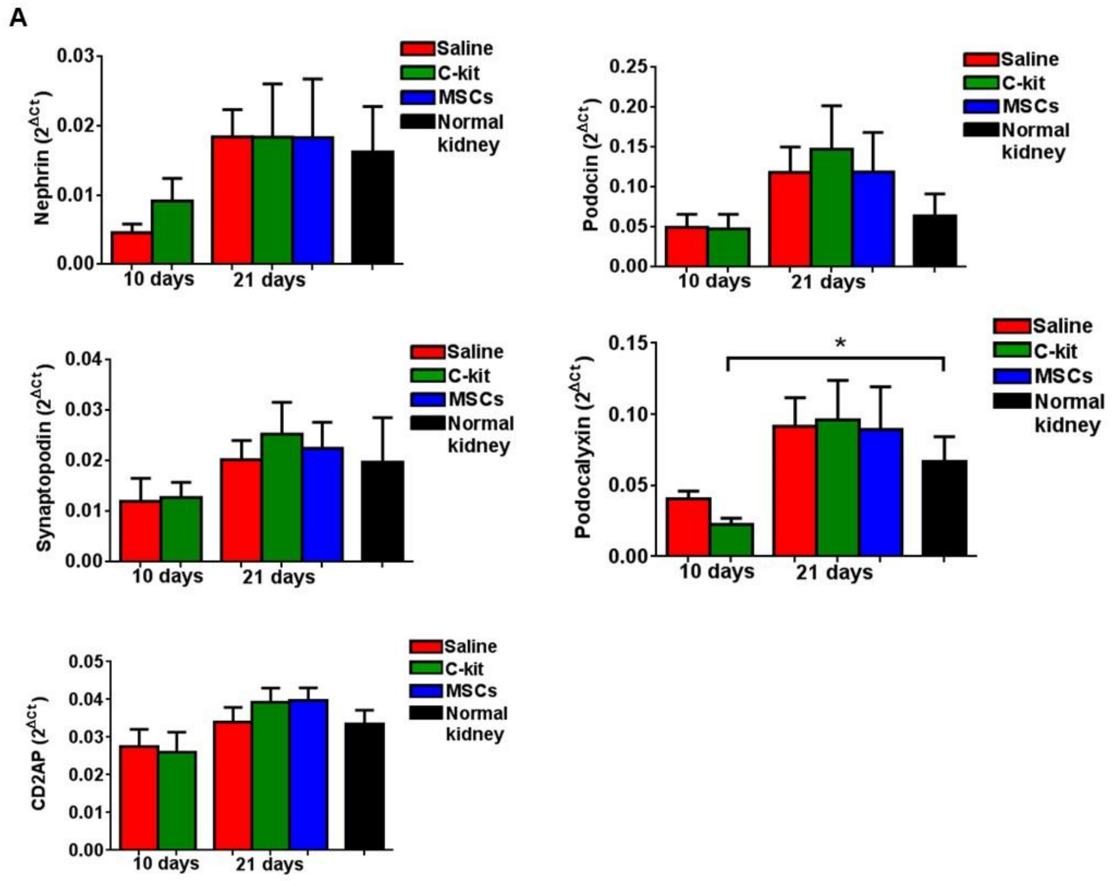

Figure 4. Gene expression after saline, c-kit^{+} progenitor/stem cell and MSCs treatment normalized to WT-1 expression.

(A) Gene expression of nephrin, podocin, synaptopodin, podocalyxin (PDX), and CD2-associated protein (CD2AP) normalized to WT-1 in kidney cortex in all three groups according to the time and compared to the normal kidney, by qPCR ($2^{\Delta Ct}$).

Error bars represent means ± SEM.

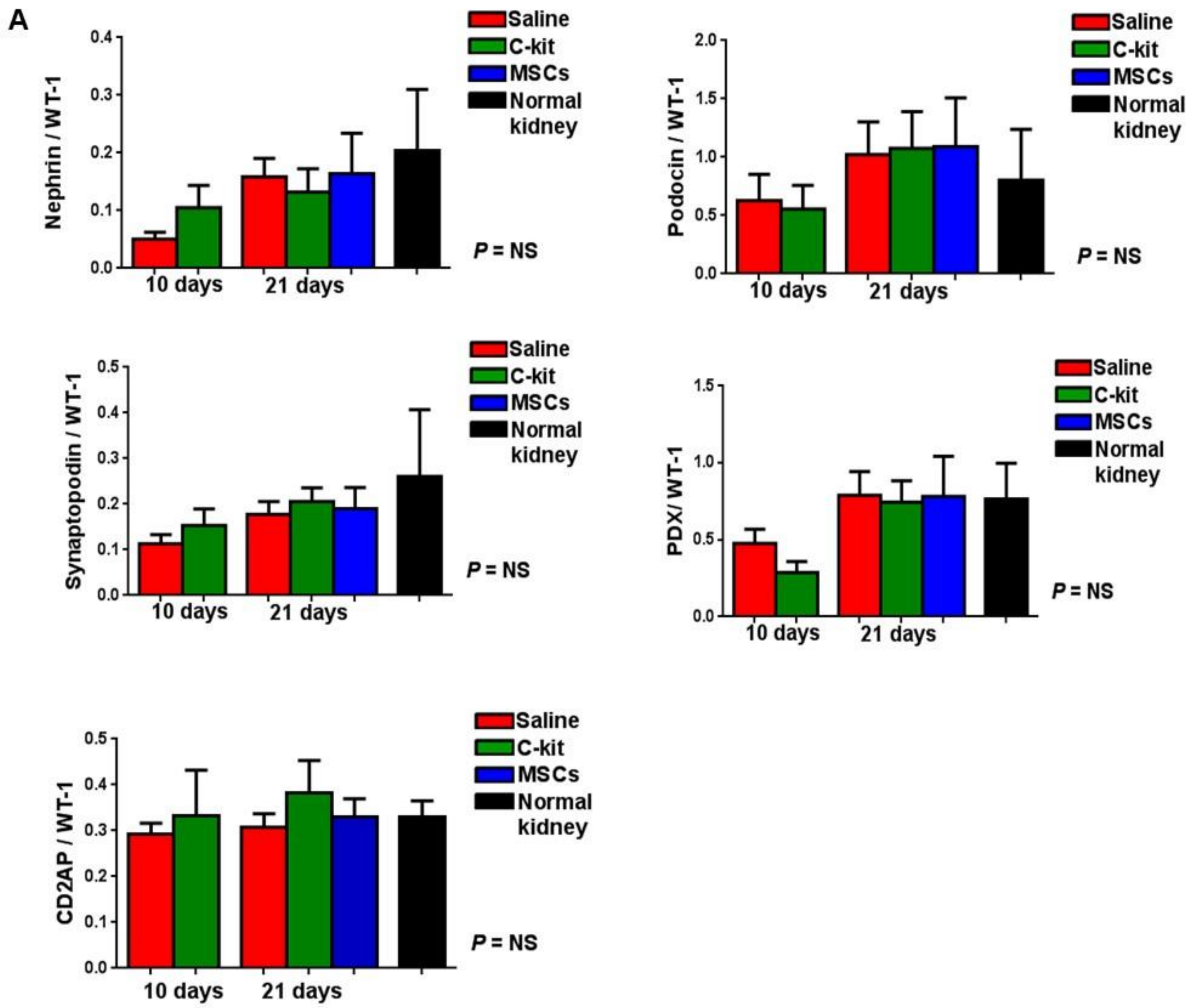

Supporting Information of Experimental Procedures

GFP (Green fluorescent protein) labeling - Lentiviral particles were grown in 293T cells. Briefly, 7x10^6 293T cells were plated in 10 cm dishes and cultured in DMEM medium containing 10% serum. Lentivirus vectors expressing green fluorescent protein was generated by cotransfection of 3 plasmids (6µg pGFP, 4µg pGagpol, and 2µg pCMV-VSVG) per plate into 293T cells. For viral packaging, Lipofectamine 2000 Transfection Reagent (Invitrogen, Carlsbad, CA, USA) was used. After overnight incubation, the culture media was replaced with fresh DMEM media containing 20% serum. Lentivirus was collected from supernatants 24h after transfection, every day, for 2 days. These supernatants were briefly centrifuged to remove cell debris, filtered, precipitated (PEG-*it* Virus Precipitation Solution 5x, System Biosciences, Mountain View, CA, USA), and frozen at -80ºC for further transduction of c-kit$^+$ cells.

C-kit$^+$ cells were plated and GFP-lentivirus was added overnight to the medium already described for c-kit cells. After that, c-kit$^+$ cells were expanded in this medium containing Puromycin 2-10 µg/ ml (Invitrogen, Carlsbad, CA, USA). Transduction efficiency was determined by analyzing the percentage of GFP$^+$ cells by FACS. Before *in vivo* experiments, we analyzed the percentage of GFP$^+$ cells and it corresponded to 93.6±1.2%.

Transmission electron microscopy - Kidney tissue slices previously fixed in Karnovsky's fixative (Electron Microscopy Sciences, Fort Washington, PA, USA). They were rinsed in three washes of buffer then postfixed overnight in 1% osmium tetroxide in 0.1M phosphate buffer. After buffer rinses, they were dehydrated through a series of graded ethanol, placed in two rinses of propylene oxide for 5 minutes each then put in a 1:1 mixture of propylene oxide: EMbed/Araldite resin (Electron Microscopy Sciences, Fort Washington, PA, USA) for overnight at room

temperature. The next day, the specimens were placed in fresh EMbed/Araldite and put in a vacuum desiccator for 2-4 hours. They were changed to fresh EMbed/Araldite and polymerized overnight in a 64^0C oven. Silver/gold sections were cut on a Leica Ultracut E (Leica, Buffalo Grove, IL, USA), stained in uranyl acetate and lead citrate, and images were captured by a Gatan Erlangshen ES1000W camera (Gatan, Pleasanton, CA, USA) in a Philips CM10 electron microscope (FEI, Hillsboro, OR, USA).
